# Soybean MADS-box gene *GmAGL1* promotes flowering via the photoperiod pathway

**DOI:** 10.1186/s12864-017-4402-2

**Published:** 2018-01-16

**Authors:** Xuanrui Zeng, Hailun Liu, Hongyang Du, Sujing Wang, Wenming Yang, Yingjun Chi, Jiao Wang, Fang Huang, Deyue Yu

**Affiliations:** 1 National Key Laboratory of Crop Genetics and Germplasm Enhancement, National Center for Soybean Improvement, Nanjing Agricultural University, Nanjing, Jiangsu 210095 China; 2 College of Agro-grass-land Science, Nanjing Agricultural University, Nanjing, Jiangsu China

**Keywords:** MADS-box gene, Flowering, Photoperiod pathway, Transgenic, Soybean

## Abstract

**Background:**

The MADS-box transcription factors are an ancient family of genes that regulate numerous physiological and biochemical processes in plants and facilitate the development of floral organs. However, the functions of most of these transcription factors in soybean remain unknown.

**Results:**

In this work, a MADS-box gene, *GmAGL1*, was overexpressed in soybean. Phenotypic analysis showed that *GmAGL1* overexpression not only resulted in early maturation but also promoted flowering and affected petal development. Furthermore, the *GmAGL1* was much more effective at promoting flowering under long-day conditions than under short-day conditions. Transcriptome sequencing analysis showed that before flowering, the photoperiod pathway photoreceptor *CRY2* and several circadian rhythm genes, such as *SPA1*, were significantly down-regulated, while some other flowering-promoting circadian genes, such as *GI* and *LHY*, and downstream genes related to flower development, such as *FT*, *LEAFY*, *SEP1*, *SEP3*, *FUL*, and *AP1*, were up-regulated compared with the control. Other genes related to the flowering pathway were not noticeably affected.

**Conclusions:**

The findings reported herein indicate that *GmAGL1* may promote flowering mainly through the photoperiod pathway. Interestingly, while overexpression of *GmAGL1* promoted plant maturity, no reduction in seed production or oil and protein contents was observed.

**Electronic supplementary material:**

The online version of this article (10.1186/s12864-017-4402-2) contains supplementary material, which is available to authorized users.

## Background

Flowering is initiated by the transition from a vegetative to a reproductive fate. In most flowering plants, this is an extremely important developmental process because it directly affects successful reproduction. Four major pathways, the photoperiod pathway, vernalization pathway, gibberellic acid (GA)-dependent pathway, and autonomous pathway, have been demonstrated to regulate flowering in *Arabidopsis thaliana* [36]. These four pathways, either alone or in combination, form a complex network that regulates plant flowering at the appropriate time based on changes in ambient conditions [8]. The four pathways are integrated by downstream target genes, including *LEAFY* (*LFY*), *FLOWERING LOCUS T* (*FT*) and *SUPPRESSOR OF CONSTANS1* (*SOC1*), with their resulting outcomes conveyed to floral meristem identity genes, such as *APETALA 1* (*AP1*), at the shoot apical meristem (SAM), which triggers the flowering process [57, 58].

Photoperiod pathways regulate flowering time by sensing changes in day length. Long-day plants, such as Arabidopsis, exhibit precocious flowering under long-day conditions but delayed flowering or non-flowering under short-day conditions [15]. In contrast, short-day plants, such as soybean and rice, display precocious flowering under short-day conditions but delayed flowering or non-flowering under long-day conditions [20]. Briefly, photoreceptors receive a light signal and transduce it to genes that are linked to the plant’s biological clock to “turn on” the circadian rhythm, which is then induced together with downstream floral organ development and flowering-time-related genes to regulate flowering time [15].

Though the genetic processes regulating flowering in Arabidopsis are well known, much less is understood about these networks in other species, especially for short-day plants with a photoperiod pathway that may differ from long-day plants [17]. Soybean (*Glycine max* [L.] Merr.) is a typical short-day plant and one of the most important commercial oil crops to the world economy, providing approximately 69% of the dietary protein and 30% of the oil consumed by humans [1]. Its reproductive growth directly affects seed yield and quality. Therefore, unravelling the genetic mechanisms underlying the regulation of flowering is of great significance to improve seed production and to study ecological adaptability in soybean. A classical genetics study revealed that ten genes, the genes *E1-E9* and J, were closely related to flowering and plant maturity in soybean [25]. Some of them have been mapped and found to be functionally involved in the photoperiod pathway and the regulation of soybean flowering [26, 31, 33, 51, 55, 58].

Genes in the MADS-box family of transcription factors (TFs) also play important roles in reproductive growth and development. They participate in the early steps of floral meristem development to specify the identity of a primordial floral organ later in flower development in *A. thaliana* [5]. However, few studies have examined soybean MADS-box genes [65]. Our group previously detected a high expression level of the *AG-like* MADS-box gene *GmAGL1* in a soybean flower cDNA microarray. Heterologous expression of *GmAGL1* in *A. thaliana* was shown to affect floral development and exhibit a premature phenotype [9].

Although studies have found that many genes are involved in Arabidopsis flowering and flower development processes [5], it has also been found that several genes do not play exactly the same role in Arabidopsis as in other crops [17]. Although the genome-wide expression of soybean MADS genes has been analysed [10, 47], soybean MADS-box genes have been much less investigated, with only a few genes functionally studied in soybeans [43]. The specific functions of most of these genes are still unknown. Because the molecular mechanism underlying *GmAGL1* remains unclear and the function of this gene in soybean has not been well studied, it is of interest to investigate the function of *GmAGL1* and elucidate its regulatory mechanism in soybean. Consequently, we transferred this gene into soybean in the present analysis and found that *GmAGL1* might be involved in plant maturation during the transition from the vegetative to the reproductive state.

## Results

### *GmAGL1* expression patterns differ in transgenic plants

The MADS-box gene *GmAGL1* (GenBank accession number: AW433203) was transformed into the soybean cultivar ‘Jack’, resulting in several *GmAGL1* overexpression (*GmAGL1-*OX) transgenic plants. Expression of *GmAGL1* was significantly elevated in most transgenic plants compared with that in wild-type (WT) ‘Jack’ (Additional file 1: Figure S1). Homozygous transgenic plants from two *GmAGL1-OX* lines (*GmAGL1*-OX line 1 and *GmAGL1*-OX line 5) were screened and used for further study.

**Fig. 1:**
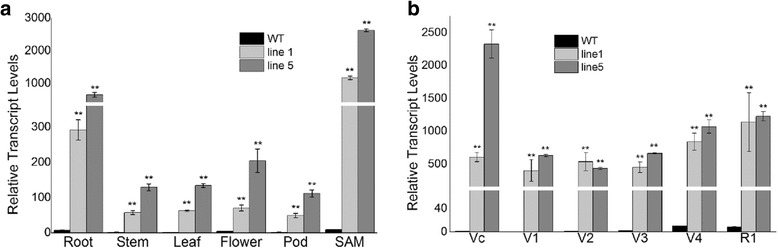
Expression patterns of the *GmAGL1* gene. **a** Gene expression of *GmAGL1* in various tissues of the five homozygous transgenic lines and WT. The T2 generations of the two homozygous transgenic lines *GmAGL1*-OX lines 1 and 5 and WT were cultured in soil. RNA was extracted from roots, stems, leaves, and shoot apical meristems (SAMs) of 20-day-old seedlings. RNA was extracted from flowers and pods of 50-day-old seedlings. WT leaves were used as a control (relative expression of 1) to compare the relative expression of other samples. **b** Gene expression of *GmAGL1* in the V0-R1 period in five homozygous transgenic plant SAMs. The T2 generations of the five homozygous transgenic lines, *GmAGL1*-OX lines 1 and 5, and WT were cultured in soil. RNA of SAMs was extracted from plants at the VC to R1 stages. VC (cotyledon) stage: single leaf half expanded, leaf margin separated; V1 (one-node) stage: single leaf fully grown, leaf margin of the first compound leaf separated; V2 (two-node) stage: the first compound leaf above the single leaf fully grown; V3 (three-node) stage: including the single leaf, three leaves on the main stem fully grown; V4 (four-node) stage: including the single leaf, four leaves on the main stem fully grown; R1 stage (the beginning of flowering): a flower is open at any position on the main stem. SAMs of WT-VC were used as a control (relative expression of 1) to compare relative expression in the other samples. *GmAGL1* transcript levels were determined by qRT-PCR. The α-*Tubulin* gene was amplified as an internal control to normalize all the data. Transcript levels were calculated using the 2^-ΔΔCt^ formula to measure the expression levels relative to *GmAGL1*. Data are presented as the means of three biological replicates, and error bars indicate SDs. Asterisks indicate a significant difference between the WT and transgenic plants (**P < 0.01, *P < 0.05)

To investigate the tissue expression patterns of *GmAGL1* in the control (WT Jack) and transgenic plants, real-time PCR was performed in roots, stems, leaves, flowers, pods and shoot apical meristem (SAM). The results showed that the expression of *GmAGL1* in both *GmAGL1-*OX transgenic lines was significantly higher than that in WT among all the tested tissues (Fig. 1a). In both transgenic lines, among all the examined tissues, expression of *GmAGL1* was highest in SAM. *GmAGL1* expression in SAM of WT and transgenic plants was then examined from the VC (cotyledon stage) to the R1 (the beginning of flowering) stage. The expression of *GmAGL1* in SAM was significantly higher in transgenic plants than in WT during the six investigated stages (Fig. 1b).

To determine whether the insertion sites would affect the expression of the *GmAGL1* gene, thermal asymmetric interlaced PCR (TAIL-PCR) was used to map the transgene insertion sites in the transgenic lines (Additional file 1: Figure S2). Interestingly, the transgene insertion sites of these two transgenic lines were only in non-coding regions; that in *GmAGL1-*OX line 1 was located in the non-coding region between the genes Glyma13G31150 and Glyma13G31160, and that in *GmAGL1-OX* line 5 was located in the non-coding region on chromosome 20, between Glyma.20G049600 and Glyma.20G049700 (these two gene models are present only in the current Wm82.a2 assembly, annotation version 1 set).

### Flowering and plant maturity in transgenic lines overexpressing *GmAGL1*

Early flowering and prematurity were observed in *GmAGL1-OX* transgenic plants.

The flowering time was measured using the measure of days after sowing (DAS) to the appearance of the first flower at any node of the main stem (R1 stage). In the field, WT Jack plants started flowering at 39.4 ± 0.49 DAS, and florescence ended at 58.6 ± 1.17 DAS, with a florescence duration of 19.2 ± 1.25 days (Table 1). Transgenic lines 1 and 5 started flowering at 36.7 ± 0.49 and 36.5 ± 0.49 DAS, respectively, approximately 3 days earlier than the WT and other lines. The whole florescence duration was 16.2 ± 0.75 and 16.1 ± 0.70 days in transgenic lines 1 and 5, respectively, approximately 3 days shorter than in the WT.

**Table 1:** Flowering time and duration of field-grown transgenic and WT soybean lines

Plant lines	Start flowering (DAS)	End flowering (DAS)	Florescence duration (Days)
*GmAGL1*-OX line 1	36.7 ± 0.49 B b	52.9 ± 0.75 B b	16.2 ± 0.75 B b
*GmAGL1*-OX line 5	36.5 ± 0.49 B b	52.6 ± 0.49 B b	16.1 ± 0.70 B b
WT	39.4 ± 0.49 A a	58.6 ± 1.17 A a	19.2 ± 1.25 A a

Data are shown as average of ten plants ± SD; different capital letters indicate a significant difference at the 0.01 level, and different lowercase letters indicate a significant difference at the 0.05 level

**Fig. 2:**
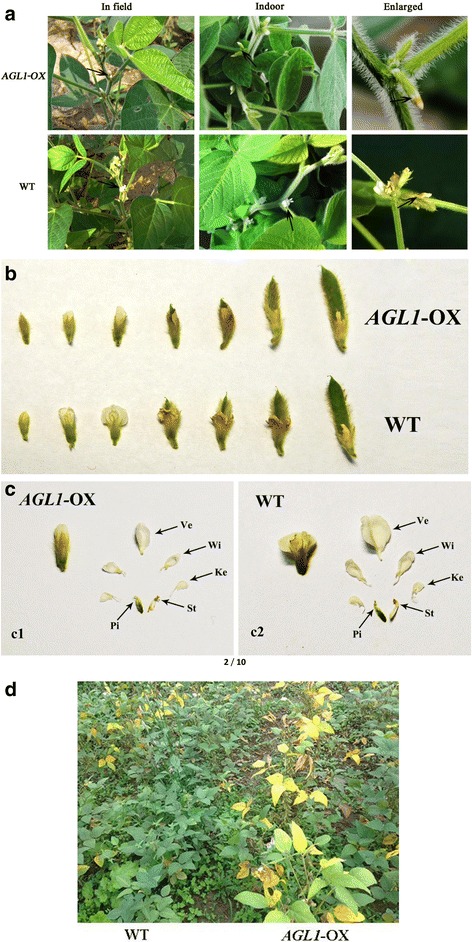
Flowering and mature phenotypes of *GmAGL1-*overexpressing plants and WT. **a** Flowers of *GmAGL1*-overexpressing plants in the field and indoors. **b** The growth state from bud to bloom and the formation of young pods of *AGL1*-OX transgenic and WT plants. **c** Anatomical observations of the flowers of *AGL1*-OX transgenic (C1) and WT (C2) plants. Ve: vexillum, Wi: wing, Ke: keel, St: stamens, Pi: pistil. **d** Differences between *AGL1*-OX transgenic and WT plants in the field

In addition to the differences in growth periods, the development of floral organs in transgenic plants was also altered. For instance, the petals of transgenic plants were not fully expanded until the soybeans were in pods and were smaller than in the WT control (Fig. 2a, b). Through anatomical observation of the floral organs at the time of flowering, the opening angle of the vexillum and wing petal was found to be smaller in transgenic plants than in WT, resulting in petals that could not fully expand. In addition to petals, the pistils and stamens of transgenic plants were also slightly smaller than in the WT control (Fig. 2c). Excluding the homozygous lines 1 and 5, the other heterozygous transgenic plants in the field undergoing the early generation also presented early flowering and incompletely expanded petals (Additional file 1: Figure S3), and they were also precocious.

Moreover, the transgenic plants showed prematurity. For example, while WT plants were still in the late filling period (R6), the leaves of transgenic plants had turned yellow and entered the early mature stage (R7) (Fig. 2d). Throughout the growing season, transgenic plants showed an average prematurity of 5–7 days compared with WT plants.

### Transgenic plant flowering under long-day/short-day conditions

**Fig. 3:**
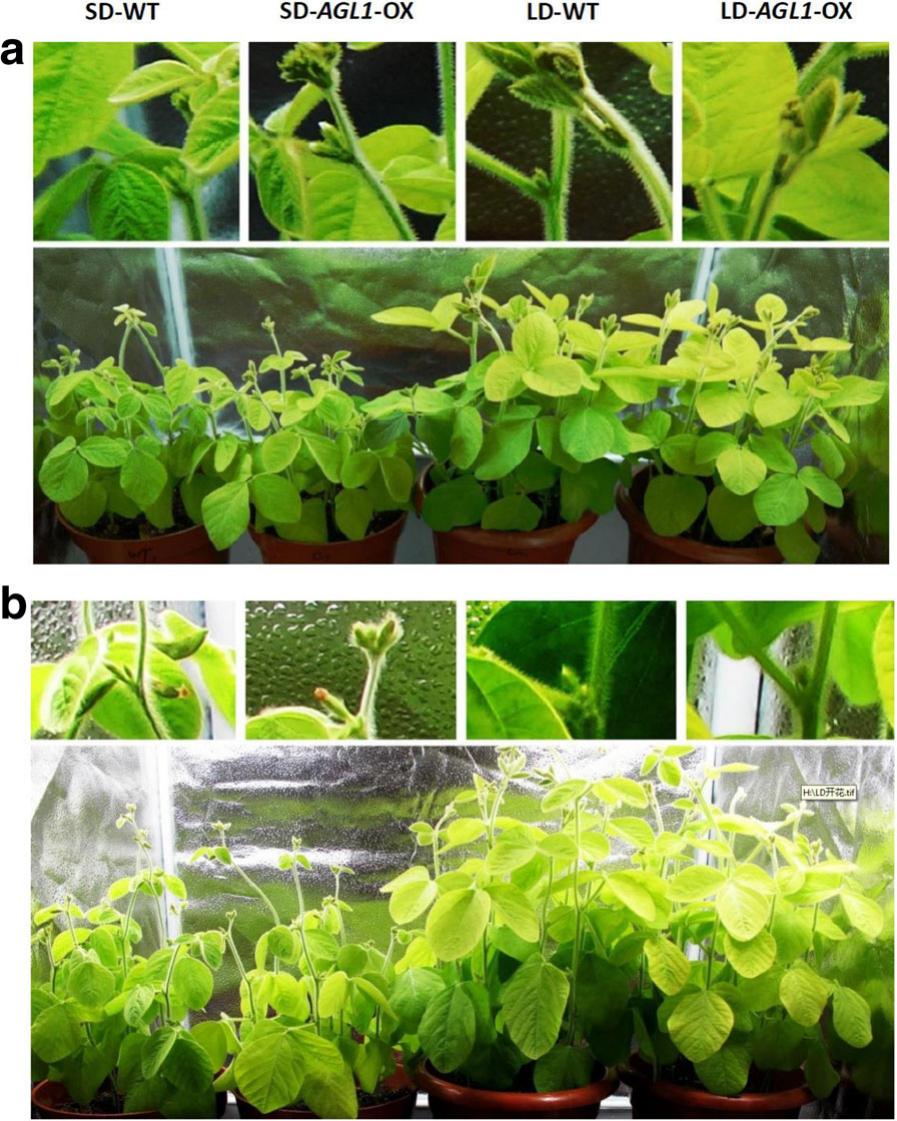
Flowering phenotypes of transgenic plants and WT under LD and SD conditions. **a** Flower development under LD and SD conditions at 40 DAS. **b** Flower development under LD and SD conditions at 52 DAS. A potted partial node is enlarged to clearly show the buds, flowers and pod fettle

As a typical short-day plant, the flowering time of soybean is strongly influenced by day length. To further investigate the effect of day length on *GmAGL1*, 7-day-old seedlings of WT and transgenic plants under 14/10-h light/dark conditions were transferred to short-day (SD, 12/12-h light/dark) or long-day (LD, 16/8-h light/dark) conditions and grown until flowering. The flowering time data showed that transgenic plants flowered precociously under both SD and LD conditions (Fig. 3). Under SD conditions, transgenic plants started flowering at 38.9 ± 0.70 and 38.6 ± 0.66 DAS, respectively, which was significantly earlier than WT (42.0 ± 0.77 DAS). However, all the soybean plants flowered later under LD than under SD conditions. Under LD conditions, transgenic line 1 and line 5 plants started flowering at 45.8 ± 0.75 DAS and 45.7 ± 0.64 DAS, respectively, which was also significantly earlier than in WT (53.6 ± 0.66 DAS). In contrast to the WT control, the early flowering phenomenon observed in transgenic plants was more significant under LD (approximately eight days) than under SD conditions (approximately three days) (Table 2).

**Table 2:** Flowering time of transgenic and WT soybean lines under short-day and long-day conditions

	Plant lines	Flowering time (DAS)
Short-day	WT	42.0 ± 0.77 C c
	GmAGL1-OX line 1	38.9 ± 0.70 D d
	GmAGL1-OX line 5	38.6 ± 0.66 D d
Long-day	WT	53.6 ± 0.66 A a
	GmAGL1-OX line 1	45.8 ± 0.75 B b
	GmAGL1-OX line 5	45.7 ± 0.64 B b

Data are shown as average of ten plants ±SD; different capital letters indicate a significant difference at the 0.01 level, and different lowercase letters indicate a significant difference at the 0.05 level

### Agronomic traits of transgenic soybean plants

To explore the effect of the *GmAGL1* gene on agronomic traits, WT Jack plants and homozygous transgenic plants in the T3/T4 (2015) and T4/T5 (2016) generations were cultured in the field and harvested in late September. No differences between transgenic and WT plants were observed for node number of the main stem, branch number per plant, seed kernel weight, total pods, total seeds, seed yield per plant, or cracked pod rate (Table 3). Regarding plant height, only the T3 (2015) generation of transgenic line 5 was dramatically shorter than the WT, whereas the transgenic line 5 plants in the T4 (2015) and T4/T5 (2016) generations showed no significant differences from WT in plant height. All the plants in transgenic line 1 also showed no significant differences from WT. *GmAGL1* may not greatly impact these agronomic traits.

**Table 3:** Descriptive statistics for agronomic traits of transgenic and WT soybean lines

		PH	NM	BR	TP	TS	SY (g)	SW (g)	CR (%)
2015	WT	44.93 ± 5.46	15.80 ± 1.40	2.50 ± 1.20	46.00 ± 12.64	80.00 ± 27.95	8.67 ± 2.99	11.19 ± 2.30	0.03 ± 0.02
	line 1 (T3)	46.00 ± 6.02	15.90 ± 0.94	1.80 ± 0.87	41.70 ± 5.44	65.70 ± 17.65	7.18 ± 2.02	11.41 ± 3.33	0.01 ± 0.02
	line 5 (T3)	36.19 ± 2.15 **	15.75 ± 0.97	2.00 ± 0.50	45.88 ± 9.61	65.88 ± 17.73	7.97 ± 1.75	12.35 ± 1.31	0.01 ± 0.02
	line 1 (T4)	40.78 ± 5.10	15.00 ± 1.00	2.88 ± 1.05	58.25 ± 10.23	108.50 ± 25.68	12.45 ± 2.87	11.63 ± 1.89	0.02 ± 0.01
	line 5 (T4)	43.76 ± 4.48	15.70 ± 0.78	2.20 ± 1.17	47.60 ± 7.17	83.00 ± 13.32	10.42 ± 2.12	12.56 ± 1.57	0.01 ± 0.02
2016	WT	51.83 ± 5.81	15.80 ± 1.08	1.30 ± 0.90	23.50 ± 5.16	37.86 ± 6.88	4.07 ± 0.43	10.67 ± 1.10	2.21 ± 0.02
	line 1 (T4)	52.90 ± 5.60	15.60 ± 1.02	1.60 ± 0.80	28.50 ± 8.05	46.30 ± 12.20	4.88 ± 0.93	10.78 ± 0.95	1.93 ± 0.02
	line 5 (T4)	52.40 ± 6.45	15.80 ± 1.40	1.30 ± 0.90	23.90 ± 5.26	38.50 ± 7.35	4.01 ± 0.54	10.73 ± 1.10	2.21 ± 0.02
	line 1 (T5)	51.60 ± 8.24	15.30 ± 0.90	1.40 ± 0.92	25.80 ± 6.24	43.80 ± 7.91	4.59 ± 0.58	10.64 ± 1.01	1.95 ± 0.02
	line 5 (T5)	50.10 ± 6.28	15.90 ± 1.30	1.30 ± 0.78	21.50 ± 4.10	35.00 ± 4.38	3.67 ± 0.28	10.61 ± 1.01	1.93 ± 0.02

Data for plant height (PH), nodes of the main stem (NM), branches (BR), total pods per plant (TP), total seeds per plant (TS), seed yield per plant (SY, measured in g), seed kernel weight (SW, measured in g) and cracked pod rate (CR measured in %) are shown as average of plants ± SD. Asterisks indicate a significant difference between the control and transgenic plants in the same year (**P < 0.01)

**Fig. 4:**
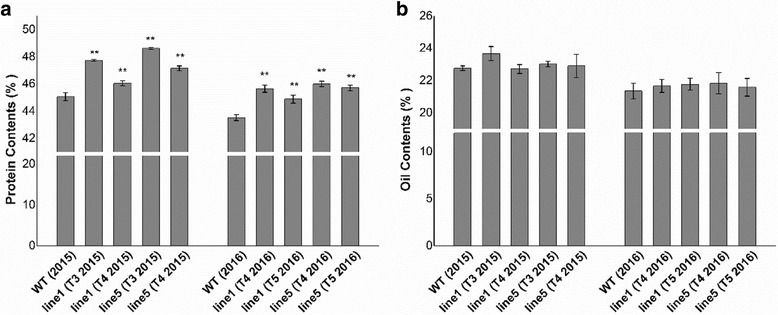
Protein and oil contents of transgenic soybean seeds. **a** The percentages of protein contents of transgenic lines and WT in the T3, T4 and T5 generations. **b** The percentages of oil contents of transgenic lines and WT in the T3, T4 and T5 generations. Asterisks indicate a significant difference between WT and transgenic plants (**P < 0.01)

Protein and oil contents are the most abundant constituents in soybean seeds, and they are also important indicators of soybean quality. By using near infrared spectroscopy, the protein, but not the oil, contents of transgenic lines in both 2015 and 2016 were found to have significantly increased (Fig. 4a) compared with WT (Fig. 4b).

### Transcriptomic differences between WT and *GmAGL1*-overexpressing transgenic plants

**Fig. 5:**
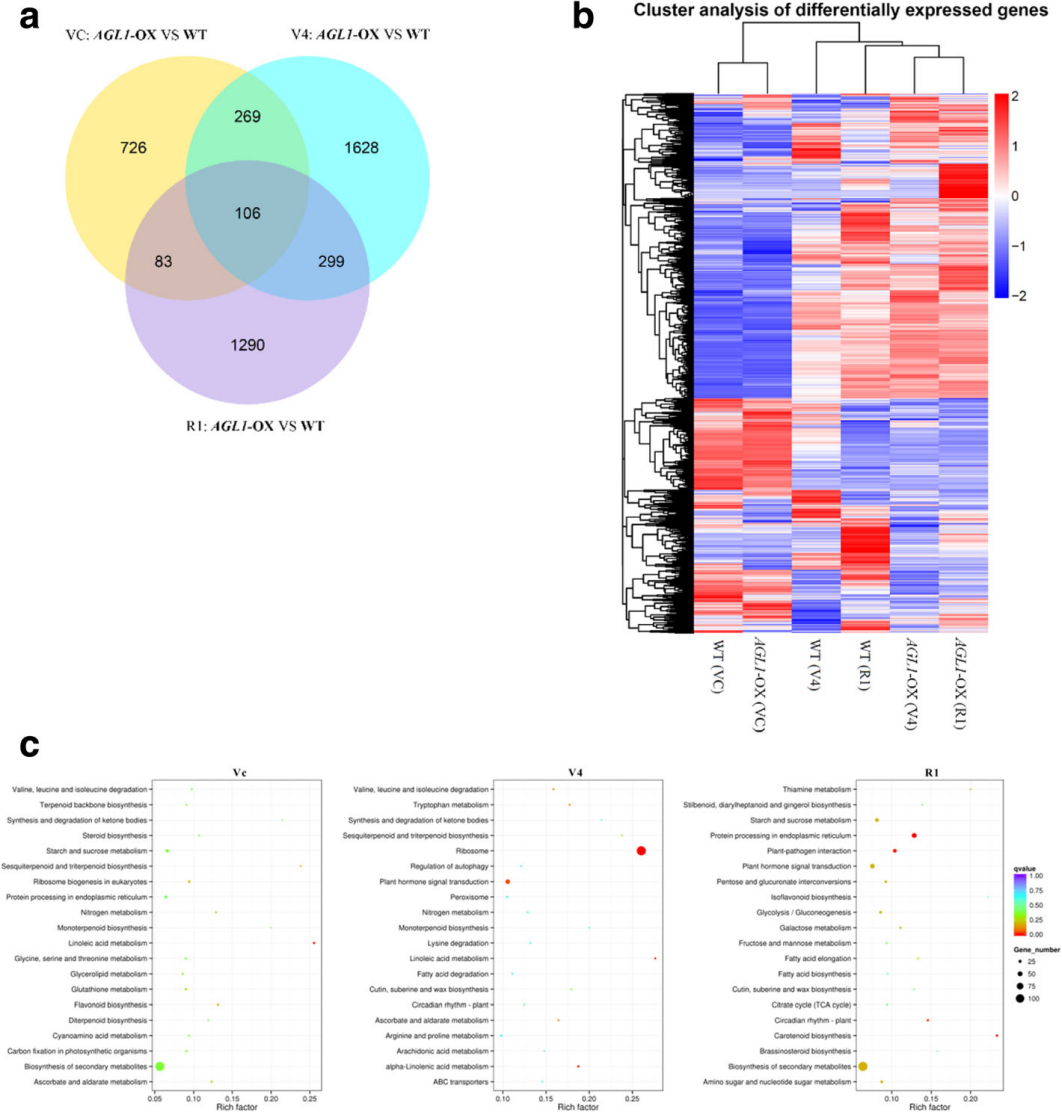
Transcriptome sequencing and cluster analysis of differentially expressed genes. **a** Venn diagram showing the number of DEGs between *AGL1*-OX transgenic and WT plants in the VC, V4 and R1 stages. **b** Heat cluster of DEGs between *AGL1*-OX transgenic and WT plants in the VC, V4 and R1 stages. **c** Scatterplot showing the most enriched KEGG pathways of DEGs between *AGL1*-OX transgenic and WT plants in the VC, V4 and R1 stages. The Y-axis shows the most enriched KEGG pathways, and the X-axis shows the enrichment factor. The sizes of the plots indicate the number of DEGs in the pathway, and the colours of the plots indicate the Q-value of each pathway

To investigate how the genetic programming is deployed in transgenic plants, Illumina RNA sequencing was applied to SAM tissues from three growth stages (VC, V4 and R1) in both transgenic and WT plants (Fig. 5a, b).

The transcriptome analysis data revealed a total of 4401 genes with significant differences in expression between the transgenic line and the WT. Among these 4401 differentially expressed genes (DEGs), 1184 genes were from the VC stage, 2302 from the V4 stage, and 1778 from the R1 stage (Fig. 5a). A total of 106 genes showed altered expressed in *GmAGL1-OX* plants in all three stages. Among them, *GmAGL1* showed the most significant changes in all three stages and was expressed at levels more than 10 times higher in transgenic plants than in controls (Additional file 2).

Cluster analysis of DEGs indicated that the gene expression pattern of transgenic plants (VC) was more similar to WT (VC) in the VC stage than in other stages (Fig. 5b). However, in the V4 and R1 stages, the expression patterns of DEGs in WT and transgenic plants were both more similar to the adjacent growth stages. Moreover, the expression pattern of DEGs in transgenic plants at V4 was more similar to WT at R1 than to WT at V4. That is, the gene expression pattern of transgenic plants in the flowering preparation state (V4) was more similar to WT plants in the flowering stage (R1), and the transgenic plants therefore exhibited earlier flowering than the WT controls.

KEGG (Kyoto Encyclopaedia of Genes and Genomes) pathway analysis of the DEGs revealed that the circadian rhythm pathway was one of the DEG-enriched pathways in the V4 and R1 stages (Fig. 5c).

### Circadian rhythm genes presented significant differences in transgenic plants

**Fig. 6:**
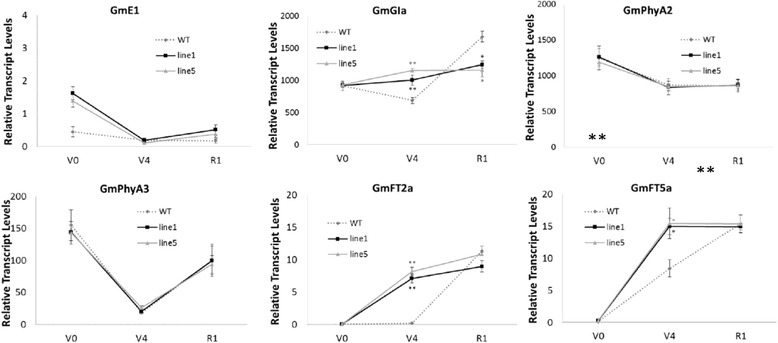
Expression of soybean maturity genes in transgenic and WT plants during three periods. The expression of genes in SAM in the VC, V4 and R1 periods in *GmAGL1*-OX line 1, line 5 and WT were assessed. Gene transcript levels were determined by qRT-PCR. The α-*Tubulin* gene was amplified as an internal control to normalize the data. Transcript levels were calculated using the 2^-ΔΔCt^ formula to determine their expression levels relative to the standard. Black squares (with a black solid line) indicate gene transcript expression in *AGL1*-OX line 1. Light grey triangles (with a light grey dotted line) indicate gene transcript expression in *AGL1*-OX line 5. Dark grey circles (with a dark grey dotted line) indicate gene transcript expression in WT. Data are presented as the means of three biological replicates, and error bars indicate SDs. Asterisks indicate a significant difference between WT and transgenic plants (**P < 0.01, *P < 0.05)

By analysing the expression of genes related to soybean flowering and maturity regulation in transgenic plants, the expression levels of the *E2* gene *GmGIa*, together with the *E7* gene *GmFT2a* and its orthologue *GmFT5a*, were found to be significantly altered in both transgenic lines compared with the WT control before flowering (Fig. 6). Soybean *E2* is orthologous to the *A. thaliana GIGANTEA (GI*) gene, which is a critical gene in the circadian rhythm [55]. *GmFT2a* and *GmFT5a* are orthologous to *AtFT*, which is also a circadian rhythm gene and plays an important role in the regulation of flowering [8].

**Fig. 7:**
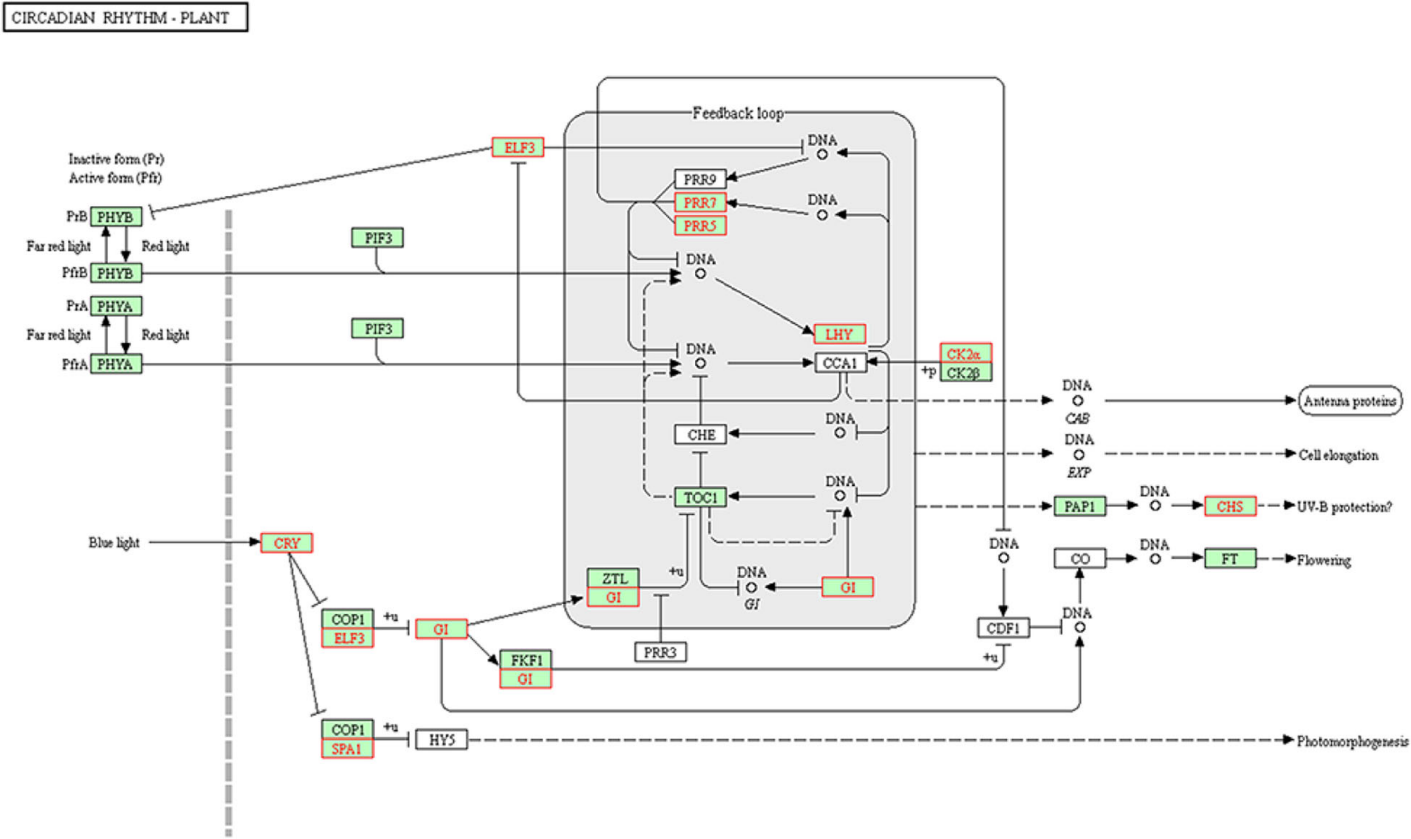
Differences in expression of circadian rhythm genes between WT and *AGL1*-OX transgenic plants. The image shows the soybean circadian clock genes in the intact biological network (http://www.genome.jp/kegg-bin/show_pathway?gmx04712). The white box indicates an Arabidopsis circadian clock gene with no homologues in soybean. The red box indicates a DEG between transgenic and WT plants. The black box indicates soybean gene expression that was not markedly changed in transgenic plants

Subsequently, we investigated the transcription of other circadian rhythm genes in the transcriptome and showed that most of the transcripts associated with soybean circadian rhythm and*/*or their orthologues differed significantly between transgenic plants and WT (soybean circadian rhythm genes were found online at http://www.genome.jp/kegg-bin/show_pathway?gmx04712 soybean circadian rhythm, Fig. 7).

### Photoperiod pathway genes presented significant differences between transgenic and control plants

**Fig. 8:**
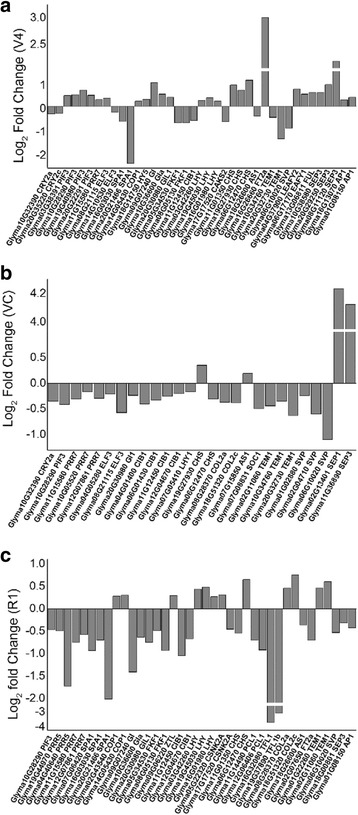
Transcripts associated with the photoperiod pathway that were regulated in *AGL1*-OX line 5. Photoperiod pathway-related genes showed alterations based on transcriptome sequencing of the VC (**a**), V4 (**b**) and R1(**c**) stages. The X-axis lists the IDs of genes that were significantly regulated in transgenic plants and their corresponding homologues in Arabidopsis. The Y-axis shows the log_2_ fold change in transcription between the transgenic plants and WT plants

Photoreceptors and circadian clocks are mechanisms for sensing and responding to the light environment via photoperiod pathways [27]. Thus, we examined the transcription of all photoperiod pathway-related genes, including photoreceptors, circadian rhythm genes and downstream flower development regulatory genes. The results showed that most of the genes associated with the photoperiod pathway in *GmAGL1*-OX line 5 were significantly differentially expressed compared with WT (Fig. 8).

SAM transcriptome sequencing data revealed that in the early vegetative stage VC, a total of 27 photoperiod pathway-related genes in transgenic plants presented differences in expression in comparison to WT. Most of the DEGs were down-regulated in the transgenic plants, whereas only four genes were up-regulated. The most highly up-regulated DEGs were the orthologues of *SEPALLATA 1* (*SEP1*) and *SEPALLATA3* (*SEP3*), which are required for the development of petals, stamens and carpels [42] (Fig. 8a).

At the V4 stage, transgenic plants showed down-regulation of 13 genes involved in the photoperiod pathway and up-regulation of 26 photoperiod genes compared with WT (Fig. 8b). The photoreceptor genes *CRY2a* and *CRY2c* and circadian rhythm genes *ELF3*, *SUPPRESSOR OF PHYA-105 1 s* (*SPA1s*), *FLAVIN-BINDING KELCH REPEAT F Box 1 s (FKF1s*), *CRYPTOCHROME-INTERACTING BASIC-HELIX-LOOP-HELIX* (*CIB1*), *CSNK2A*, and *AS1* homologues, together with the flowering regulatory genes *TEMPRANILLO 1 s* (*TEM1s*) and *SHORT VEGETATIVE PHASE* (*SVP*) homologues showed slightly decreased expression in transgenic plants. Other circadian genes that showed increased expression included *PIF3s*, *CHSs*, *CONSTITUTIVE PHOTOMORPHOGENIC1 (COP1*), *LHYs* and *GIs*, together with the flowering and flower development-related genes *FT* orthologous gene *FT2a*, *LFYs*, *FRUITFULL (FUL*), *AP1s*, and *SEP3s*. Among them, the genes showing the greatest differences in expression were the critical flowering-promoted gene *FT2a* and the flower development-related gene *SEP3*.

During flowering (R1 period), 26 genes related to the photoperiod pathway showed down-regulated expression in transgenic plants compared with WT (Fig. 8c). Most of the circadian rhythm genes, including the *GIs*, were up-regulated once in the V4 stage and then down-regulated after flowering. The flowering regulatory genes *PCLs*, *AS1*, *TFL1s*, *FT2a*, *SVP*, *SEP3*, and *AP1* were also down-regulated in transgenic plants. Among them, the most differentially expressed genes were two orthologues of *TFL1*, which regulate inflorescence meristem development [46]. In contrast, 12 genes were slightly up-regulated.

During the transition from vegetative growth to reproductive growth, the expression levels of photoreceptor genes and most circadian rhythm genes seemed to be lower in transgenic than WT plants. In transgenic plants, the circadian rhythm and flower development genes, including *GIs*, *FT2a*, *SEPs*, and *AP1*, showed increased transcript levels when ready to blossom, followed by a sharp decrease after flowering.

### Gene expression changes in other flowering regulatory pathways

The main flowering pathways in *A. thaliana* are the photoperiod, vernalization, GA and autonomous pathways. Investigating gene expression in other pathways, in addition to the photoperiod pathway, is also worthwhile.

Expression of homologues of the *A. thaliana* vernalization pathway-related genes *REDUCED VERNALIZATION RESPONSE 1* (*VRN1*), *REDUCED VERNALIZATION RESPONSE 2* (*VRN2*) [60], *VERNALIZATION 5* (*VRN5*) [14], *EARLY FLOWERING 7* (*ELF7*), *EARLY FLOWERING 8 (ELF8*) [18], and *FRIGIDA-ESSENTIAL 1* (*FES1*) [45] was detected. The results showed that only four of the 41 detected genes were down-regulated significantly: the *AtVRN5* homologue Glyma07G09800, the two *AtVEL1* homologues Glyma13G00920 and Glyma17G07000 in the V4 stage, and one *AtELF8* homologue, Glyma09G07980, in the R1 stage. Expression of the remaining genes was not significantly different from WT (Additional file 1: Table S4).

The critical gene in the autonomous pathway in *A. thaliana* is *FLOWERING LOCUS C* (*FLC*) [6]. FLC-like genes from plants other than Brassicaceae have not yet been reported, and *FLC* homologues have not been found in the soybean gene database phytozome server (https://phytozome.jgi.doe.gov/pz/portal.html#!info?alias=Org_Gmax) or in the Soybase server (https://soybase.org/GlycineBlastPages/). However, the genes that interact with *A. thaliana FLC* have been studied. *PHOTOPERIOD-INDEPENDENT EARLY FLOWERING 1* (*PIE1*) [39], *FRIGIDA* (*FRI*) [45], *VERNALIZATION INDEPENDENCE* (*VIPs*), *FLOWERING TIME CONTROL PROTEIN* (*FCA*), *FY* [32], *FLOWERING LOCUS D* (*FLD*), *FVE* [4], *LUMINIDEPENDENS (LD*) and *FPA* genes [19] all interacted with *FLC* in *A. thaliana* and are expressed in both soybean and Arabidopsis. Research has further revealed that these genes are also involved in the regulation of flowering time. Therefore, we investigated the transcription of these genes. A total of 40 *FLC-*interacting genes were detected in all three stages, and only four genes showed significantly changed expression in the transgenic plants: the *AtFVE* homologue Glyma15G02770, which was down-regulated in the VC stage; the *AtFCA* homologue Glyma07G36630, which was down-regulated in the V4 stage; and one *AtFPA* homologue, Glyma13G42061, which was up-regulated in the V4 stage; and the *AtFPA* homologue Glyma11G13490, which was down-regulated in the R1 stage. The expression of the remaining genes did not show significant differences from WT (Additional file 1: Table S5).

In the GA pathway, one *AtRGL3* homologue, Glyma03G37851, was significantly down-regulated in the VC stage compared with WT. The expression levels of nine genes changed significantly in the V4 stage. Glyma20G34260, with homology to *AtGAI*; Glyma10G02790, Glyma02G17010, and Glyma03G30460, with homology to the GA-related gene *OsGID1B* (*GA INSENSITIVE DWARF1B*); and Glyma20G37430, with homology to *OsGID1C* (*GA INSENSITIVE DWARF1B*) [3, 37], were up-regulated, and Glyma02G01530, Glyma06G23940 and Glyma10G33380, with homology to *AtRGA1*, were down-regulated. Moreover, one *AtRGL1* homologue, Glyma18G43580, was significantly down-regulated in the R1 stage. The expression of the remaining genes was not significantly different from WT (Additional file 1: Table S6).

### MADS-box gene transcription and translation changes in transgenic plants

**Fig. 9:**
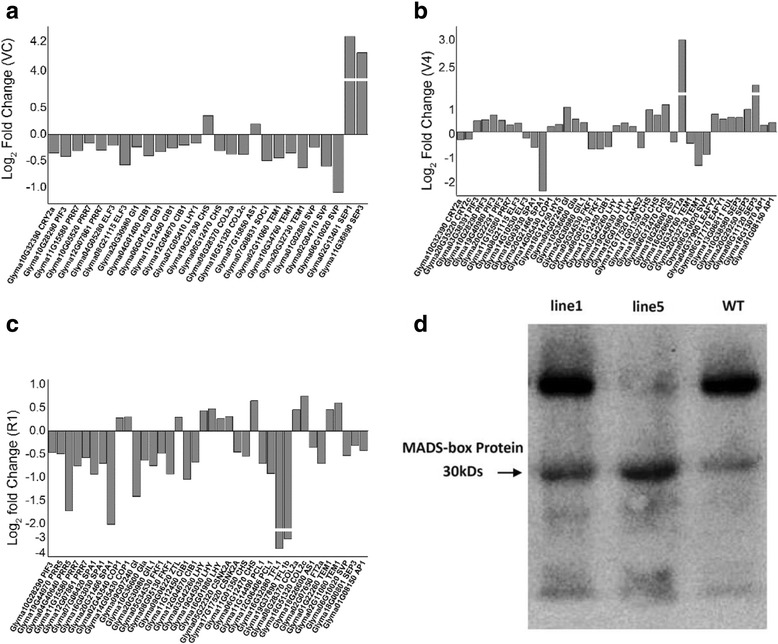
Differentially expressed MADS-box genes and MADS-box protein expression in WT and transgenic plants. **a-c** MADS-box genes showed alterations based on transcriptome sequencing of the VC (**a**), V4 (**b**) and R1 (**c**) stages. The X-axis lists the IDs of MADS-box genes that were significantly regulated in *AGL1-*OX plants and their corresponding homologues in Arabidopsis. The Y-axis shows the log_2_ fold change in transcription between the *AGL1*-OX transgenic plants and WT. **d** MADS-box protein immunoblot analysis of the two *GmAGL1*-OX lines (1 and 5) and WT. Both transgenic lines presented higher contents of MADS-box proteins than WT plants at 30-kDa

MADS-box genes play important roles in flower development, including floral meristem identity, the maintenance and promotion of flower meristem development, and flowering and maturity [49]. Thus, we comprehensively investigated the transcription of MADS-box genes using the RNA-sequencing data. In the three stages (VC, V4 and R1), a total of 132 MADS-box transcripts were detected in WT and transgenic plants (Additional file 1: Table S3). The expression of 34 genes differed significantly between transgenic and WT plants. In addition to *GmAGL1*, 15 MADS-box genes were up-regulated in one of the three stages, with one gene showing increased expression in two stages, 13 genes showing decreased expression in one stage, and three genes showing decreased expression in two stages. However, only *GmAGL1* gene expression was significantly increased in all three periods (Fig. 9a–c). Interestingly, *GmAGL1* was the most significantly highly expressed gene among all the genes detected in this study (more than a ten-fold change (log2) at the transcript level).

To further confirm this result, Western blotting was performed, revealing higher MADS-box protein contents in SAM from transgenic plants than from WT plants (Fig. 9d).

## Discussion

In this study, *GmAGL1* was transferred into the soybean variety ‘Jack’, and the transgenic plants presented an early flowering phenotype. In addition, the floral features were reduced. Gene insertion site analysis indicated that these phenotypes were caused by the overexpression of *GmAGL1*, not by the insertion of exogenous fragments that inactivate endogenous genes. Transcriptome sequencing analysis of WT and *GmAGL1* overexpression plants indicated that *GmAGL1* may promote flowering mainly via the photoperiod pathway. This discovery likely reveals a new function of the *AGL* gene to promote flowering and maturity.

### Roles of the MADS-box gene in flower development and plant maturity

Genes in the MADS-box family of transcription factors (TFs) play important roles in reproductive growth and development, including flowering and fruit development. For example, the MADS-box genes *SOC1* (*AGL20*) [21], *AGAMOUS-LIKE15* (*AGL15*) and *AGAMOUS-LIKE18* (*AGL18*), along with *SVP* (*AGL22*) and *AGAMOUS-LIKE24* (*AGL24*) [11], are involved in regulating plant flowering regulation, and, in addition to *AP1* (*AGL7*), the MADS-box genes *FUL* (*AGL8*) [23], *CAULIFLOWER* (*CAL*, *AGL10*) [12], *SEPALLATA* genes [41] and *SEEDSTICK* (*STK*, *AGL11*) [35] also participate in flower identity determination, floral organogenesis, and fruit and seed development. MADS-box genes have been well studied in many plants, but not in soybean, and the specific functions of most genes in this species are unclear.

In this study, *GmAGL1*-overexpressing transgenic lines presented early flowering, early maturity, and a smaller floral organ, both indoors and in the field, when compared to WT plants. Early in the vegetative stage (VC), with *GmAGL1* was up-regulated in transgenic plants, the transcript levels of the floral organ identity MADS-box genes *SEP1* and *SEP3* were also highly up-regulated in transgenic plants, which may be beneficial to the formation of flowers and the floral organ identity in transgenic plants. The soybean cultivar ‘Jack’ generally blossomed in the V5-V6 stage. During the flowering preparation period (V4 stage), the MADS-box genes *FT2a*, *SEP3, AP1,* and *FUL*, along with the additional flowering regulatory gene *LFY*, were up-regulated in transgenic plants compared with WT plants, which may be beneficial to the formation of flowers and promote flowering in transgenic plants. In the R1 stage, the reduction of the *FT2a* and floral organ identity genes *SEP3* and *AP1* might have been the major cause of the undersized development of the floral organ in transgenic plants. *SHP*, *AP1*, *AP3*, *SEP1*, *CAL*, *AG*, *AGL44*, *AGL6*, *PI* and *AGL8* are *AG-like* MADS-box genes, which have been shown to regulate early flower development [17, 34]. Our previous research has shown that *GmAGL1* is a nuclear-localized transcription factor and can interact directly with SEP-like proteins in soybean flowers. It was also found to be sufficient to activate the expression of *A. thaliana* ALC, IND, STK, SEP1, and SEP3 [9]. *GmAGL1* may play a role in flower development together with other AG-like MADS-box genes. Thus, while the flower-development-related MADS-box genes *FT2a*, *SEPs*, and *AP1* were down-regulated in the R1 stage, this did not affect the ability of the transgenic plants to form complete floral organs with high expression levels of *GmAGL1*.

The role of the *GmAGL1* gene in seed development was also investigated, and, with the exception of precocity, there were no phenotypic differences between the seeds of the transgenic plants and the controls, although the storage protein content was increased in *GmAGL1-*overexpressing plants.

### *GmAGL1* promotes flowering mainly via the photoperiod pathway

It is generally accepted that the photoperiod pathway, vernalization pathway, GA-dependent pathway, and autonomous pathway are the four major pathways regulating flowering in *A. thaliana*. Photoperiodicity promotes flowering by combining internal circadian rhythms and external environmental signals. Plant photoreceptors and circadian rhythms integrate external signals and transmit the signal via a central oscillator [15]. Although key photoperiod genes have been studied in many species, research on soybean photoperiod-related genes is not as well established. Thus, *A. thaliana* and other plants were used as references in this study to identify photoperiodic regulation-related genes in soybean. Gene expression between *GmAGL1-*OX transgenic plants and WT before flowering was compared, and the results showed that the *GmAGL1* gene may affect flowering primarily through the photoperiod pathway.

Cryptochromes are the photolyase-related blue-light receptors that regulate plant’s responses to light and/or the circadian clock in all major evolutionary lineages [44]. During the transition from the vegetative to reproductive phases, the biological circadian clock genes *PRR5*, *PRR7*, *SPA1*, *FKF1*, and *CIB1* act on the downstream gene *CRY2* to affect the flowering process ([38, 56]). Compared with the control, most of the circadian clock genes, such as *PRRs*, *ELF3* and *CIB1*, exhibited decreased expression in *GmAGL1-*OX transgenic plants (Figs. 7 and 8a, b). A relationship between the decreased expression of *ELF3* and an insensitivity to photoperiod with regard to floral initiation has been characterized in *A. thaliana* [63]. A similar effect may also occur in soybean, leading to a reduced requirement for a short-day rhythm in transgenic plants to induce reproductive growth. Early flowering of transgenic plants under both short-day and long-day conditions (Fig. 3, Table 2) further confirmed this hypothesis.

Throughout the photoperiod pathway, most genes act similarly to their roles during the blooming of Arabidopsis and tobacco. The flowering-time-promoted genes *CHS* [53], *COP1* [54], *LHY* [61], *GI*[55], *FT* [22], *COL2* [52], and *LFY* [7], together with the flowering-identity and floral-organ-development genes *SEP1*, *SEP3*, *FUL*, and *AP1* [48], were all highly expressed in transgenic plants compared to WT plants, while the flowering-inhibited genes *SPA1* [27], *SVP*, *TFL1* (ShannonandMeeks-Wagner, 1991) were down-regulated and led to the promotion of blooming in transgenic plants. Previous research in our laboratory has shown that GmAGL1 can interact directly with soybean and *A. thaliana* SEP1 and SEP3 proteins [9], and therefore, high expression levels of *GmAGL1* seem to enhance the roles of SEP1 and SEP3 and thus promote flowering. During the transition from vegetative growth to the flowering stage, photoperiodic DEGs formed a complete flowering regulatory pathway that included photoreceptors, the circadian rhythm centre, and downstream regulatory genes (Additional file 1: Figure S4). In addition to the GA-dependent pathway, gene expression in the flowering regulation vernalization pathway and autonomous pathway showed little influence on the entire flowering-regulated pathway. In addition to being a flower-promoting substance, GA is an important signalling hormone that may be involved in other important biological processes [62]. Therefore, we inferred that the photoperiod pathway was the main mechanism by which *GmAGL1* promoted flowering.

In conclusion, the photoperiod pathway is the major mechanism by which *GmAGL1* promoted soybean flowering. *GmAGL1-*OX seemed to weaken the demand for strict short-day circadian rhythms in soybeans. Altering the expression patterns of a series of genes associated with optical signals and circadian rhythms, and that further affect the flower morphology, allowed the transgenic plants to undergo a rapid transition to the flowering stage induced by a shorter short-day photoperiod. GA might also play a role in the promotion of flowering.

### There is no significant effect of the overexpression of *GmAGL1* on seed production

As the *GmAGL1* homologue *SHP1/2* has the effect of promotes cracking of pods in *A. thaliana* [28] and as heterologous expression of *GmAGL1* in *A. thaliana* also increases the number of pods cracked [9], we detected the cracking rate of soybean pods in this experiment. To our surprise, the results showed that neither the cracking rate nor the mature and immature pod phenotype of transgenic plants was significantly different from WT (Table 3; Additional file 3). This phenomenon may be due to differences between plant species and fruit types, suggesting that the function of this gene is not identical in the two plant species.

It is generally accepted that cultivated soybean is self-fertile and self-pollinating with an outcrossing rate of less than 4% [16]. Soybeans do not rely on bright petals and fragrance to attract pollinators. Although the petals of transgenic plants may be smaller and not fully expanded (Fig. 3a–c), this type of undersized development is not a major factor affecting soybean seed production.

*GmAGL1-OX* transgenic plants exhibited precocious flowering both in the field and indoors under short-day/long-day conditions and showed prematurity of at least five days in the field compared with the control. These photoperiod responses may be useful for developing short-season cultivars because early maturity provides agricultural producers with more time to harvest and prepare the next crop in the crop rotation for agricultural production. In many crops, seed production is affected by a shortened period of vegetative growth and precocity. In this experiment, the agronomic traits of four generations of transgenic plants in 2015 and 2016 were observed. In 2016, due to the droughts during the drum stage, most of the plants suffered a serious decrease in production. As a result, there were many under-filled seeds, which led to deviations in yield, and oil and protein contents, but there was no significant difference in agronomic traits between transgenic and WT plants (Table 3). We hypothesize that transgenic plants may compensate for the energy required for fruiting organs by reducing consumption by vegetative organs, leading to reduced vegetative growth of stems and leaves (reduced plant height) and slightly reduced growth of genital organs (petals) without affecting normal development. Therefore, despite the shortened florescences of transgenic plants, there was no significant reduction in the seed yield of transgenic lines. The finding that the protein content of transgenic plants was increased without affecting the yields indicated that the shortened growth cycle represents an advantageous characteristic of transgenic plants.

## Conclusions

In this work, a MADS-box gene, GmAGL1, was overexpressed in soybean. Phenotypic analysis showed that *GmAGL1*-overexpressing lines not only resulted in early maturation but also promoted flowering and affected petal development. Further, the role of *GmAGL1* was shown to be much more significant in promoting flowering under long-day conditions than under short-day conditions. Transcriptome sequencing analysis of *GmAGL1*-overexpressing plants indicated that *GmAGL1* may promote flowering mainly via the photoperiod pathway. Interestingly, while overexpression of *GmAGL1* promotes plant maturity, the results did not show reductions in seed production or oil and protein content.

## Methods

### Plant materials

Seeds of the soybean (*Glycine max* [L.] Merr.) cultivar ‘Jack’ were provided by the National Center for Soybean Improvement (Nanjing, China). Soybean seedlings were grown under natural conditions in the isolation plots of Jiangpu Experimental Station, Nanjing Agricultural University, Jiangsu, China. Seeds were sown in late June and harvested in late September to early October from 2013 to 2016. The first generation of *GmAGL1-*overexpressing (*GmAGL1-*OX) transgenic plants (T1) grew in 2013, the second generation of transgenic plants (T2) grew in 2014, the third/fourth generation of transgenic plants (T3/T4) grew in 2015, and the fourth/fifth generation of transgenic plants (T4/T5) grew in 2016. T4 seeds were harvested from T3 plants that had been grown in a chamber (CONVIRON AP100, Winnipeg, Canada) under a 14/10-h (day/night) photoperiod at 30/20 °C (day/night) with a relative humidity of 60 ± 5% and an average light intensity of 1100 μmol/m^2^/s from Nov 2014 to Mar 2015.

All the indoor experimental T2 and T3 plants in this study were cultured in chambers (CONVIRON AP100, Winnipeg, Canada) under a temperature of 30/20 °C (day/night) with a 60 ± 5% relative humidity, average light intensity of 1100 μmol/m^2^/s, normal photoperiod of 14/10 h (light/dark), long-day photoperiod of 16/8 h (light/dark), and short-day photoperiod of 12/12 h (light/dark).

Wild-type (WT) ‘Jack’ was grown and used as a control for every experiment in this paper.

### Generation of transgenic soybean plants

Full-length *GmAGL1* (Gm14g273100) was amplified by PCR using gene-specific primers designed based on sequence information obtained from the NCBI database (Additional file 1: Table S1). PCR conditions were chosen according to the polymerase manufacturer’s protocol (Phanta™ Super-Fidelity DNA Polymerase, Vazyme). The *GmAGL1* sequence was then cloned into the plant binary vector pmG610021 under the control of the CaMV35S promoter (Additional file 1: Figure S5). The resulting vector containing the glyphosate resistance gene *cp4-EPSPE* was introduced into *Agrobacterium tumefaciens* strain EHA105 by electroporation and prepared for transformation via the cotyledon-node method [40]. Shoots germinated from the cotyledon and eventually regenerated a complete plant. The experimental process and the growth of transgenic plants during the different periods are shown in Additional file 4.

Glyphosate-resistant transgenic tissue culture seedlings were confirmed by PCR analysis (primers are listed in Additional file 1: Table S1). Then, Taq-Man probes for *cp4-EPSPE* were used to detect whether the transgenic plants were homozygous according to the manufacturer’s instructions (ABI 7500 Real-Time PCR System, Applied Biosystems, Carlsbad, CA, USA). Gene-specific primers were designed using Primer 5.0 software (Primer-E Ltd., Plymouth, UK), and Taq-Man probes were designed using NTI vector software (Invitrogen Life Technologies). All the primers and probes are described in Additional file 1: Table S1. Homozygous *GmAGL1-*OX transgenic plants in the T3 and T4 generations were used for further experiments.

### Preparation of genomic DNA, RNA and cDNA

Genomic DNA was extracted from the leaves of WT and *GmAGL1-*OX transgenic plants using the modified CTAB method [2] and then used for transgene detection by PCR and T-DNA insertion flanking sequence TAIL-PCR analyses.

Total RNA was extracted using TRIzol reagent (Invitrogen, CA, USA) according to the manufacturer’s instructions. Leaves of WT and *GmAGL1-*OX transgenic plants (T1) were collected to analyse the *GmAGL1* expression level, and roots, stems, leaves, flowers, pods, and shoot apical meristem (SAM) of WT and *GmAGL1-*OX transgenic lines (T3) were collected to analyse the tissue expression patterns of *GmAGL1.* Roots, stems, leaves, and shoot apical meristem (SAM) were collected from 20-day-old plants; flowers and immature pods were collected from 50-day-old plants (10–15 days after flowering); SAMs from VC to R1 stage were collected for the analysis of temporal expression patterns of *GmAGL1*. All the above tissues were frozen immediately in liquid nitrogen and stored at −80 °C until further use. The extracted RNAs were then used to prepare cDNA. cDNA was synthesized from 2 μg of RNA in a 10 μl reaction volume using the Prime Script 1st Strand cDNA Synthesis kit (TaKaRa, Shiga, Japan).

### TAIL-PCR and flanking DNA sequence analysis

T-DNA insertion flanking sequences from transgenic plants were isolated from genomic DNA using the thermal asymmetric interlaced PCR (TAIL-PCR) method as described by Liu and Whittier [29]. The gene-specific primers used for TAIL-PCR were designed using Primer 5.0 software (Primer-E Ltd., Plymouth, UK) and are described in Additional file 1: Table S1.

PCR products were separated by 1% agarose gel electrophoresis (agarose-L, Nippon Gene Co., Ltd., Toyama, Japan; electrophoresis apparatus), and then, the sequences that were longer than 2 kb were sequenced by the Sequencing Services Company (Beijing Liuhehuada Genomics Technology Co., Ltd. Shanghai, China). The sequencing data were analysed using the NCBI Blast (http://www. ncbi.nlm.nih.gov/BLAST/), Phytozome (http://phytozome.jgi.doe.gov/pz/portal.html), and SoyBase (http://www.soybase.org/GlycineBlastPages/) servers to confirm their genomic locations.

### Real-time quantitative PCR

Real-time quantitative PCR was performed by the SYBR Green method on an ABI 7500 Fast Real-Time PCP system (Applied Biosystems, Carlsbad, CA, USA) using Aceq qPCR SYBR Green Master Mix (Vazyme Biotech Co, Nanjing, China). The following procedure was used for qRT-PCR: 1 cycle at 95 °C for 5 min, followed by 40 cycles at 95 °C for 30 s, 60 °C for 30 s, and 72 °C for 30 s. The gene expression data were analysed using the 2^-ΔΔCt^ method as described by Livak and Schmittgen [30] (LivakandSchmittgen, 2001). The primers used in these experiments are listed in Additional file 1: Table S1.

### Flowering time and flowering phenotype

Flowering time was measured from sowing to the R1 stage, with the appearance of the first flower at any node of the main stem. In this experiment, five different transgenic lines and one wild-type plant were chosen. In the field, 10 plants were randomly screened to determine the flowering time of each replicate, and three biological replicates were assessed. In the indoor experiment, each line was germinated using 15 seeds per pot under 14/10-h day/night conditions for one week, and then 10 seedlings with a generally consistent growth trend were left for further observations. Each pot was considered one replicate, with a total of three replicates of 10 plants per transgenic line/WT transferred to short-day (12/12-h light/dark) and long-day (16/18-h light/dark) conditions, respectively.

The flowers of WT and *GmAGL1-*OX transgenic line 5 with the highest expression were selected and dissected to observe the floral organ phenotype. Flowers and young pods of the *GmAGL1*-OX line 5 and WT in the R3 stage (beginning podding) were chosen and photographs obtained to compare growth.

### Investigation of agronomic traits

To investigate soybean growth and harvesting, eight traits were measured: the plant architecture traits plant height (PH), branch number per plant (BR), node number of the main stem (NM), and cracked pod rate (CR) and the yield components total pods per plant (TP), total seed number per plant (TS), seed yield per plant (SY, measured in g), and kernel weight (SW, measured in g). For the measurement of CR, soybean pods from each plant were placed in a paper bag (10 × 15 cm) and baked at 37 °C for four days, and then, the number of cracking pods was counted for the CR measurements. The cracked pod rate per plant was equal to cracking pod number per plant/total pod number per plant × 100% (SR) [13]. The other seven traits were measured as previously described by Zhang et al. [64].

Three replicates of 10 individuals from WT and two *GmAGL1*-OX transgenic lines were screened for all eight trait measurements.

The protein and oil contents of the soybean seeds were measured by scanning the near infra-red (NIR) absorption spectrum of the seeds using the Grain Analyser (Infratec™ 1241, FOSS Analytical AB, Denmark). This instrument has an extended wavelength range of 570–1100 nm of NIR, and it scans and analyses 10 sub-samples of input seeds and provides a recorded reading of the protein and oil contents of soybean seeds according to the instrument built-in computational model: NIR Spectroscopy model S0090711 Soybeans Dry Weight. Triplicate samples of each line were measured.

Experimental data were statistically analysed using SPSS 14.0 software. The t-test was applied to compare transgenic lines and WT.

### Protein extraction and western blotting

A total of 100 mg of SAM from WT and *GmAGL1*-OX l transgenic line 1 and line 5 was used to extract total protein with the ProteoPrep Total Protein Extraction Sample Kit (Sigma-Aldrich, St. Louis, MO, USA) according to the manufacturer’s instructions. The MBP-MADS antibody was prepared by Vazyme Biotech Co, Ltd. (Nanjing, Jiangsu, China). The immunogenic recombinant fusion protein MBP-MADS expressed in *E. coli* had the following amino acid sequence: MGRGRVELKRIENKINRQVTFAKRRNGLLKKAYELSVLCDAEVALIIFSNRGKQYEF.

Western blotting was performed as described by Yamaji and Ma [59]. Briefly, 20 μg of total protein was separated by 10% SDS-PAGE at 80 V for approximately 3 h and then transferred onto a PVDF membrane. The membrane was blocked with BSA for 2 h and then hybridized with anti-MADS antiserum for 10 h (overnight). The protein was detected with BCIP/NBT and placed in the dark for approximately 10 min until colour development occurred.

### Transcriptome sequencing and analysis

cDNA library preparation, sequencing, and preliminary analysis of RNA-sequencing data were performed by Beijing Novogene Institute at Shanghai (Shanghai, China). cDNA libraries were constructed from SAM samples of WT and *GmAGL1*-OX line 5 at the VC, V4 and R1 stages. The samples were prepared using the Illumina TruSeq RNA sample Prep kit (Illumina, Inc., San Diego, CA, USA) and sequenced on the Illumina HiSeq™ 2000 platform (Illumina, Inc., San Diego, CA, USA). Gene expression was analysed using the FPKM (expected number of Fragments Per Kilo base of transcript sequence per Millions base pairs sequenced) method [50]. To calculate fold changes, the number of reads for each gene in each library was normalized to the total number of mapped reads for the library, and direct ratios (log2) were calculated between the different developmental stages. Transcripts with a significant *P* value (<0.05) were considered to be differentially expressed. Additionally, KEGG (Kyoto Encyclopedia of Genes and Genomes) pathway analysis of DEGs was performed at http://www.kegg.jp/ for active gene pathways [24].

## Additional files

Additional file 1: Figure S1. Relative *GmAGL1* expression in the leaves of the transgenic lines. **Figure S2**. Transgene integration sites in five homozygous transgenic lines. **Figure S3**. Flowers of *GmAGL1* overexpression lines in the field. **Figure S4**. The overexpression pathway of *GmAGL1* promotes flowering in soybean. **Figure S5**. Vector pmG610021, which includes *GmAGL1.* **Table S1**. Primer sequences used in detecting transgenic lines. **Table S2**. Taq-Man assay results of transgenic plants in the T1 generation. **Table S3**. Transcripts of all the detected MADS-box genes. **Table S4**. Transcripts associated with the vernalization pathway. **Table S5**. Transcripts associated with the autonomous pathway. **Table S6**. Transcripts associated with the GA-dependent pathway. (PDF 1269 kb)

Additional file 2: Transcriptome data. (XLSX 10026 kb)

Additional file 3: Phenotype of immature pods. (PDF 427 kb)

Additional file 4: The growth of transgenic plants in different periods. (PDF 560 kb)

## Abbreviations

AGL: AGAMOUS-LIKE
AP1: APETALA1
CAL: CAULIFLOWER
CIB1: CRYPTOCHROME-INTERACTING BASIC-HELIX-LOOP-HELIX
COP1: CONSTITUTIVE PHOTOMORPHOGENIC1
DAS: Days from sowing
DEGs: Different expression genes
FCA: FLOWERING TIME CONTROL PROTEIN
FKF1: FLAVIN-BINDING KELCH REPEAT F Box 1
FLC: FLOWERING LOCUS C
FT: FLOWERING LOCUS T
FUL: FRUITFULL
GA: Gibberellins acid
GI: GIGANTEA
KEGG: Kyoto Encyclopedia of Genes and Genomes
LD: Long day
LFY: LEAFY
OX: Overexpression
SAM: Shoot apical meristem
SD: Short day
SEP1: SEPALLATA 1
SEP3: SEPALLATA3
SOC1: SUPPRESSOR OF CONSTANS1
SPA1: SUPPRESSOR OF PHYA-105 1
STK: SEEDSTICK
SVP: SHORT VEGETATIVE PHASE
TF: Transcription factors
WT: Wile-type
