# Supplementary material for: Soybean MADS-box gene GmAGL1 promotes flowering via the photoperiod pathway
Source: BMC Genomics. 2018 Jan 16;19:51. doi: 10.1186/s12864-017-4402-2 (PMC5769455; doi:10.1186/s12864-017-4402-2)
Supplement: Supplementary file 1 — Relative GmAGL1 expression in the leaves of the transgenic lines. Figure S2. Transgene integration sites in five homozygous transgenic lines. Figure S3. Flowers of GmAGL1 overexpression lines in the field. Figure S4. The overexpression pathway of GmAGL1 promotes flowering in soybean. Figure S5. Vector pmG610021, which includes GmAGL1. Table S1. Primer sequences used in detecting transgenic lines. Table S2. Taq-Man assay results of transgenic plants in the T1 generation. Table S3. Transcripts of all the detected MADS-box genes. Table S4. Transcripts associated with the vernalization pathway. Table S5. Transcripts associated with the autonomous pathway. Table S6. Transcripts associated with the GA-dependent pathway. (PDF 1269 kb) [file 12864_2017_4402_MOESM1_ESM.pdf]

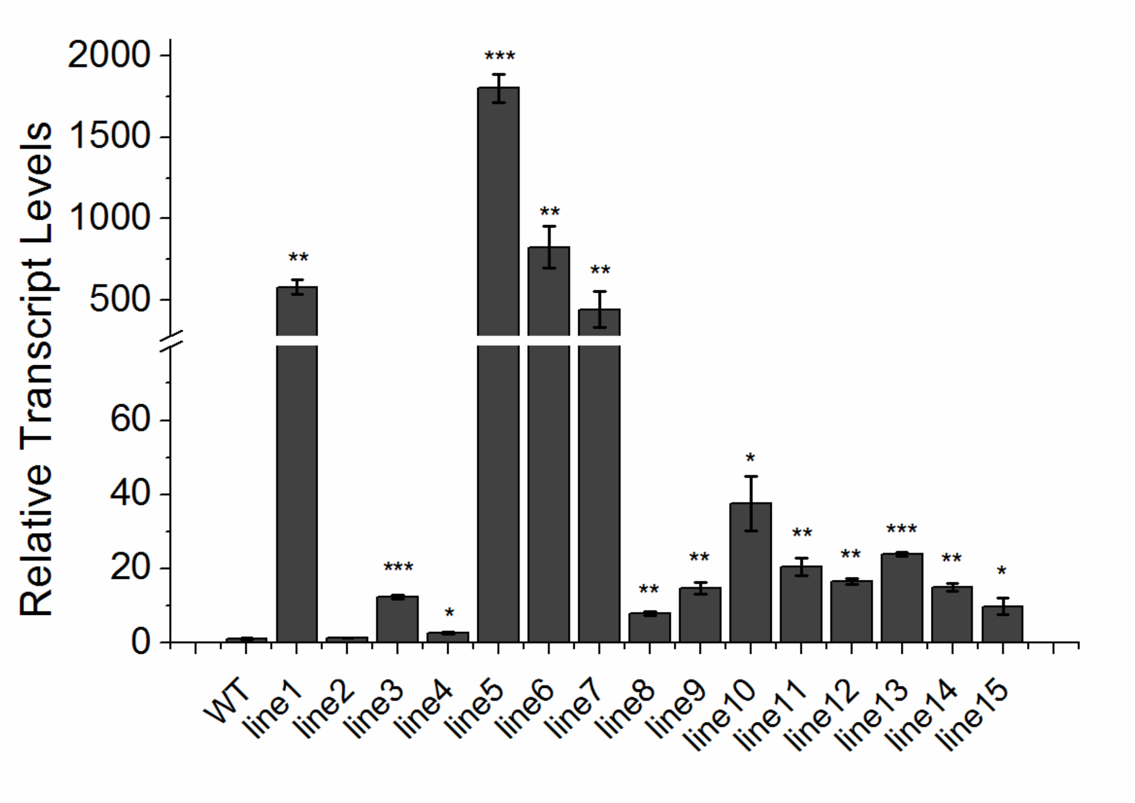

Supporting Fig. 1 Relative *GmAGL1* expression in the leaves of the transgenic lines

T1 generations of 15 transgenic lines and WT were cultured in soil. RNA was extracted from the leaves of 20-day-old seedlings. *GmAGL1* transcript levels were determined by qRT-PCR. Theamplificationα-*Tubulin*genewasusedasaninternalcontroltonormalizeallthedata.Transcript levels were calculated using the formula $2^{-\Delta\Delta Ct}$ for the expression levels relative to *GmAGL1*. Leaves of WT was used as control (Relative expression is 1) to compare the relative expression of other samples. Data represent means of three biological replicates with error bars indicating SD. Asterisks indicate a significant difference between the control and treated samples (*P < 0.05，**P < 0.01，***P < 0.001).

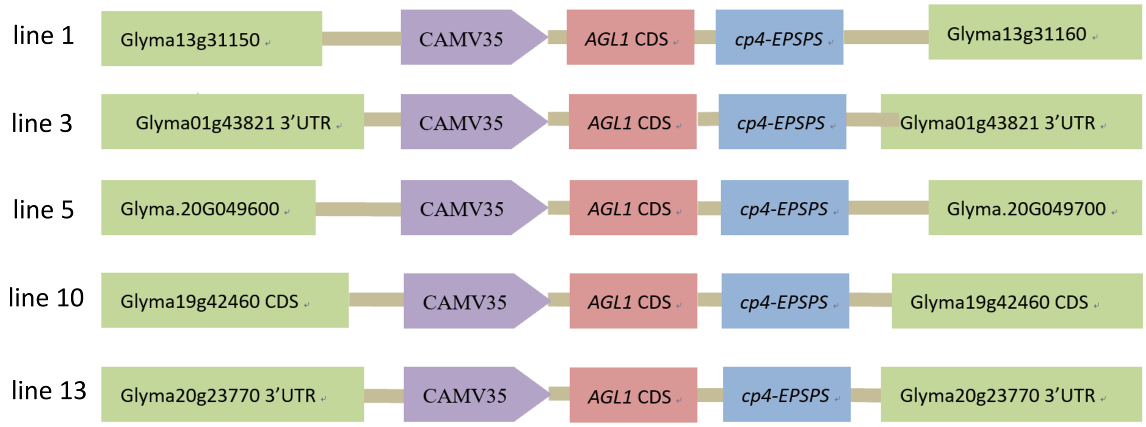

Supporting Fig. 2 Transgene integration sites in the five homozygous transgenic lines

Transgene insert sequence (containing a CaMV35S promoter, the *GmAGL1* CDS and the resistance gene *cp4-EPSPS*) is about 2300bp. All the sequencing sequences obtained were blasted on the phytozome server and the soybase server to determine the insertion position. In transgenic line 1, the transgene insert sequence inserted into the long non-coding region between Glyma13g31150 and Glyma13g31160. In transgenic line 3, the transgene insert sequence inserted into the 3'UTR of a Myb-like DNA-binding domain Glyma01g438213. In transgenic line 5, the transgene insert sequence inserted into the long non-coding region between Glyma.20G049600 and Glyma.20G049700, and these two gene models are present only in the current Wm82.a2 assembly, annotation version 1 set. In transgenic line 10, the transgene insert sequence inserted into the CDS of a Histone-like transcription factor (CBF/NF-Y) and archaeal histone Glyma19g42460. In transgenic line 13, the transgene insert sequence inserted into the 3'UTR of Glyma20g23770 which is a PF01535 - PPR repeat.

Supporting Fig. 3 Flowers of *GmAGL1* over expression line 6 and line7 in filed

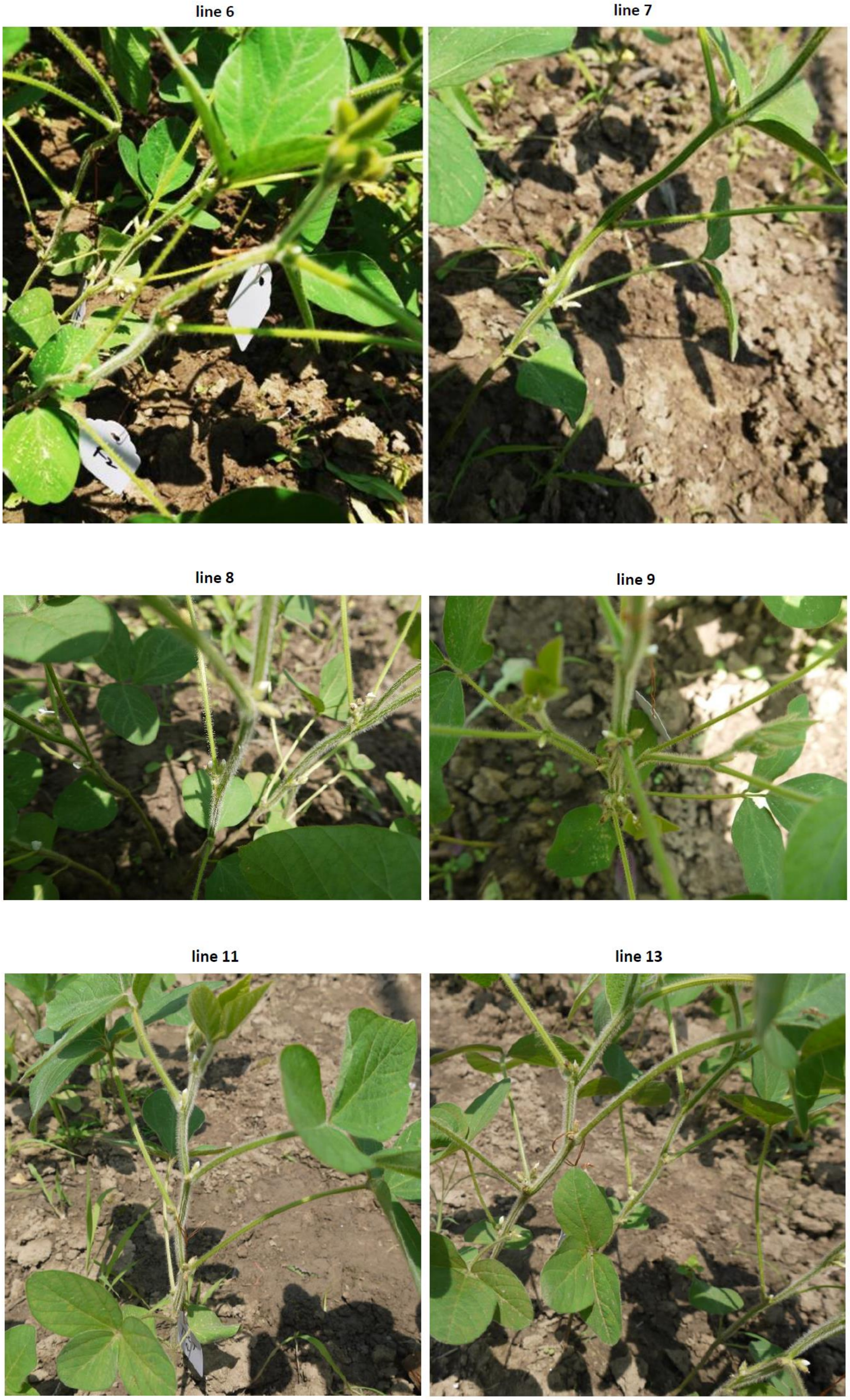

Supporting Fig. 4 The pathway of over expression *GmAGL1* promote flowering in soybean

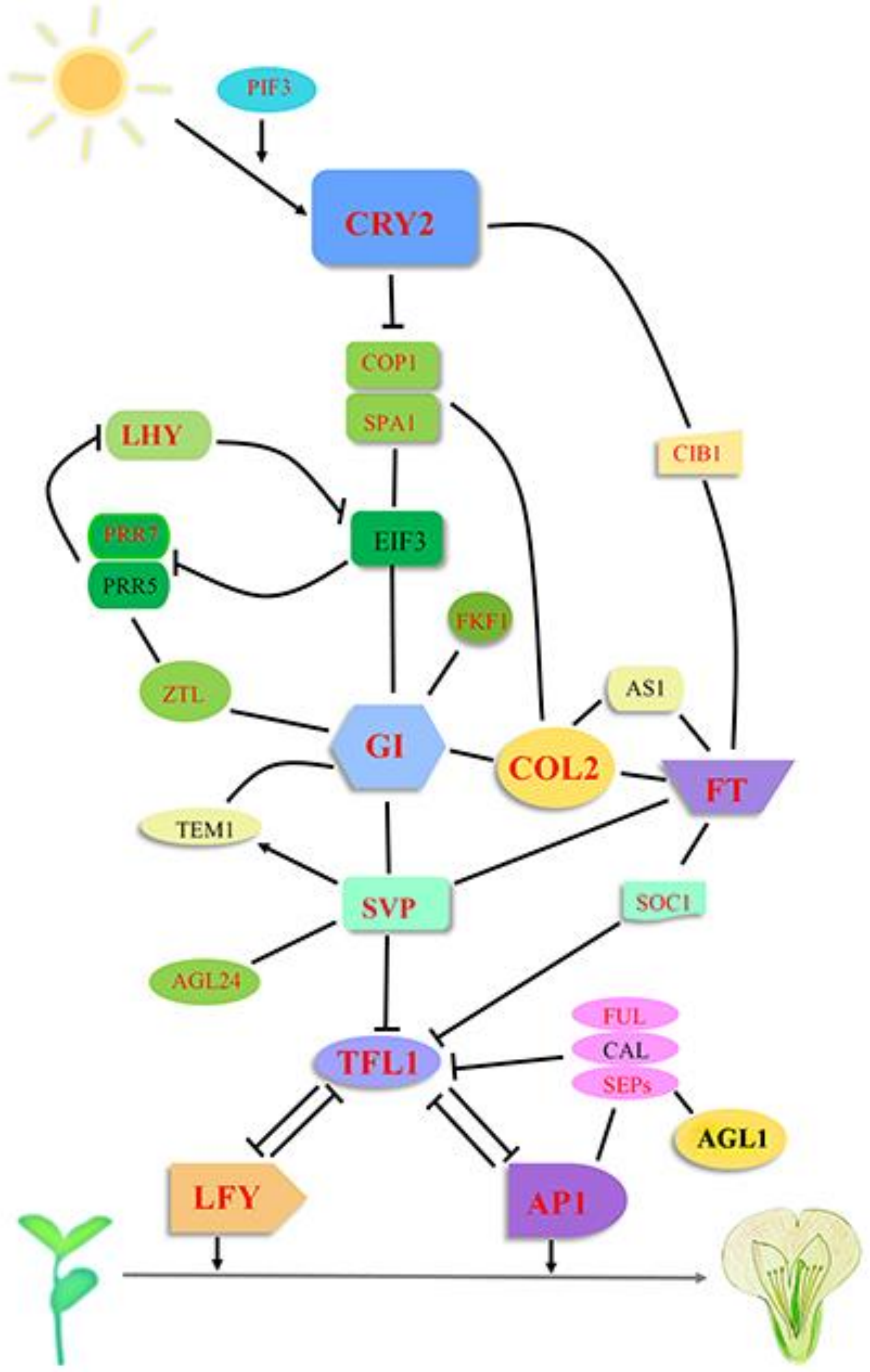

Arrows indicate a promoting, T-ends indicate an inhibiting genetic interaction. straight line indicate an interaction without a known direction. The red font indicates that the genes had already been identified or functional studies in soybean. Black font indicates that the genes were Arabidopsis or other plants homologous genes which had not been identified or functional studies in soybean yet.

Supporting Fig.5 Detailed diagram of vector pmG610021 including *GmAGL1*.

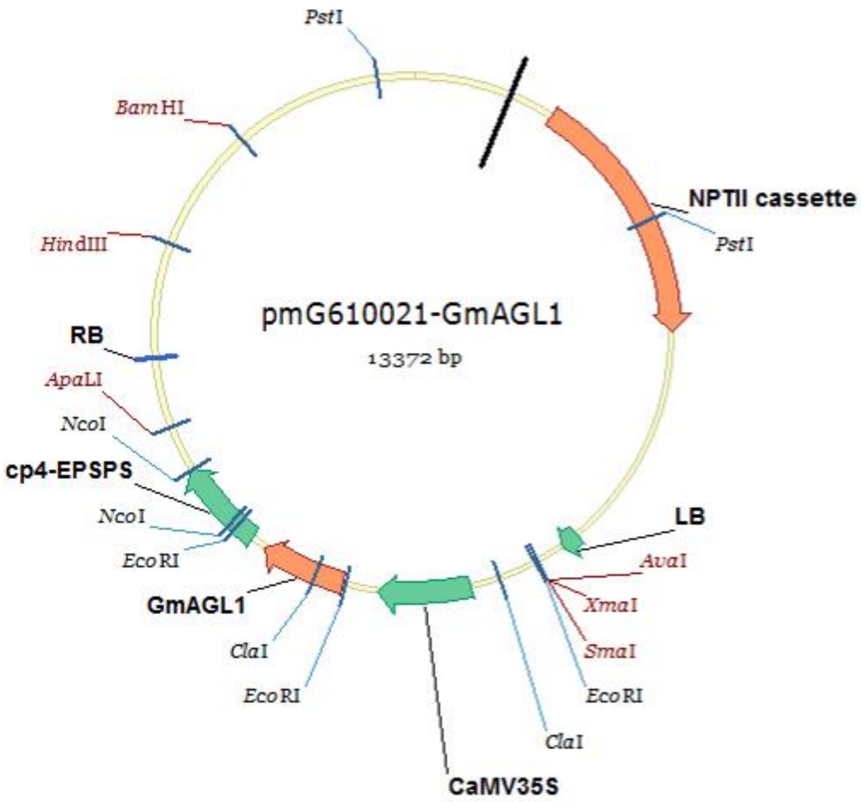

Supporting Table 1. Primer sequences used in transgenic detected

Name	Sequence	Used
AGL1-R	TCAGCAGGAAGCGTCCAAAT	*GmAGL1* expression
AGL1-F	TAATCATCGCAAGACCGGCA	*GmAGL1* expression
EPS-R	CCTTCGCANGACCCTTCCTCTATA	*cp4-EPSPS* Taq-Man
EPS-F	ATCCTGGCGCCCATGGCCTGCATG	*cp4-EPSPS* Taq-Man
EPS-P	TGCCGGTCTTGCGATGATT	*cp4-EPSPS* Taq-Man Probe
TR1	ACAGTGTTAGAGGAACGATCGATAGG	Tail-PCR Right-specific primer1
TR2	GCTCTCTGAGTCTGAAGGAATTAAAGAACCTGG	Tail-PCR Right-specific primer2
TR3	TGCGAGTCAACCATACCTCCACAATC	Tail-PCR Right-specific primer3
TL1	CAGACTCAGAGAGCTAAGAGCTTCAC	Tail-PCR Left-specific primer 1
TL2	CTATCGATCGTTCCTCTAACACTGTTGTTGGCA	Tail-PCR Left-specific primer2
TL3	CTTCTGTGAACACCCTTCTGGTATTGCTTCG	Tail-PCR Left-specific primer3
E1-R	TCCAACATGAGCAACCCTTCA	*GmAGL1* expression
E1-F	AGGCTTCGCATATGGTGGATT	*GmAGL1* expression
GIa-R	AGCTTGCTTCACGCCTACTT	*E2 gene GmGIa* expression
GIa-F	CTCTTGGCCTCTTGCTTCCA	*E2 gene GmGIa* expression
PhyA3-R	GTGAGCCCCTAGGCATTGTT	*E3 gene GmPhyA3* expression
PhyA3-F	CCCTAATCGCCACACCTTGT	*E3 gene Gm PhyA3* expression
PhyA2-R	TGAGCCAGTCAAGCCGTATG	*E4 gene GmPhyA2* expression
PhyA2-F	GCTCCCACTAGGCAACGATT	*E4 gene GmPhyA2* expression
FT2a-R	ACCGTGTATGCACCAGGATG	*E7 gene GmFT2a* expression
FT2a-F	CAGCAACTGGCAATCCAAGG	*E7gene GmFT2a* expression
FT5a-R	GGGCGATTAGCAATGGCTTG	*E8 gene GmFT5a* expression
FT5a-F	GTTGCTAGGGCTAGGTGCAT	*E8 gene GmFT5a* expression

Supporting Table 2. Taq-Man assay results of transgenic plants in T1 generation

Plant ID	Copy level	Plant ID	Copy level	Plant ID	Copy level
line1-2	Low	line5-8	Med	line9-6	Low
line1-4	Low	line5-9	Med	line10-2	Med
line1-7	Med	line6-1	Low	line10-7	Med
line2-3	Low	line6-2	Low	line10-9	Low
line2-6	Low	line6-6	Low	line11-2	Low
line2-8	Low	line7-1	Low	line11-3	Low
line3-1	Med	line7-2	Low	line12-1	Low
line3-3	Low	line7-4	Low	line12-2	Low
line3-4	Low	line8-2	Low	line13-1	Low
line4-1	Low	line8-4	Low	line14-1	Low
line4-5	Low	line8-6	Low	line15-1	High
line4-6	Low	line9-3	Med	WT1	0
line5-2	Low	line9-5	Low	WT2	0

Copy level Low indicate a single copy insert; copy level Med indicate a double copy insert; copy level High indicate multiple copies of the insert; WT is the negative control copy level is 0.

Supporting Table 3. Transcripts of all the detected MADS-box gene

GENE ID	At Ortholog	VC		V4		R1	
		log2FC	P value	log2FC	P value	log2FC	P value
Glyma01G02525	AGL21	-2.1229	0.1340	-3.5453	0.0035	-1.1314	0.3540
Glyma01G02880	AGL22, SVP	-0.2397	0.0235	-0.0034	1.0000	0.2346	0.1521
Glyma01G08130	AGL2, SEP1	Inf	0.9928	0.2002	0.6478	-0.1142	0.5819
Glyma01G08150	AGL2, SEP1	0.1243	1.0000	0.3796	0.0296	-0.4258	0.0433
Glyma01G37470	AP3, ATAP3	0.7440	0.2981	0.3229	0.1248	-0.3662	0.0207
Glyma02G04710	AGL22, SVP	-0.5969	0.0000	-0.1588	0.1406	-0.0135	0.9464
Glyma02G13401	AGL2, SEP1	4.2327	0.0048	0.3274	0.1977	0.0661	0.7313
Glyma02G13420	AGL7, AP1	-0.5292	1.0000	0.2348	0.1954	-0.2578	0.0663
Glyma02G30990	AGL62	0.8825	0.5755	-0.4786	0.6907	-0.8637	0.4595
Glyma02G33040	AGL18	-0.9533	0.0999	0.2809	0.3500	0.0625	0.7997
Glyma02G35080	AGL62	Inf	0.6811	NA	NA	Inf	0.9911
Glyma02G38091	AGL16	-0.0613	0.9692	-0.3574	0.6132	0.4307	0.3954
Glyma02G45730	AGL5, SHP2	-0.2152	1.0000	0.5180	0.4814	-0.5328	0.2371
Glyma03G02191	AGL20, SOC1	0.7302	0.6911	-0.1736	0.2276	-0.1096	0.4225
Glyma03G02210	AGL6	-Inf	1.0000	-0.1821	0.5709	-0.7512	0.0000
Glyma03G19880	AGL80, FEM111	0.4672	0.7387	-0.8398	0.1983	-1.0581	0.2596
Glyma03G26261	AP3, ATAP3	NA	NA	Inf	1.0000	Inf	0.9911
Glyma04G02980	AP3, ATAP3	-0.7315	0.7218	0.4822	0.4656	-0.1836	0.2381
Glyma04G04641	AGL66	NA	NA	1.1905	0.5530	-2.0699	0.0093
Glyma04G31847	AGL8, FUL	0.0222	1.0000	0.2481	0.2494	-0.0621	0.6684
Glyma04G42420	PI	NA	NA	0.2137	0.4619	-0.0450	0.8123
Glyma04G43640	AGL11, STK	0.4989	0.7304	1.1913	0.4848	-0.5884	0.3356
Glyma05G00960	AGL65	Inf	0.9928	-0.7611	0.8230	-0.3070	1.0000
Glyma05G03660	AGL19, GL19	-1.8488	0.0013	-0.3764	0.0014	-0.1820	0.2584
Glyma05G07286	AGL3, SEP4	-Inf	0.7023	0.3957	0.2305	-0.3390	0.0727
Glyma05G07380	AGL8, FUL	-0.7191	0.5773	0.1949	0.0793	-0.1666	0.4319
Glyma05G28130	AGL70, FCL3, MAF3	-0.7203	0.0046	-0.1136	0.4163	-0.0740	0.6786
Glyma05G28140	AGL9, SEP3	1.6856	0.0672	0.2853	0.4961	-0.3462	0.1892
Glyma05G29595	AG	-1.5993	0.7770	0.7119	0.1476	0.3736	0.1379

Glyma05G35820	AGL29	NA	NA	3.0974	0.1325	-0.3487	1.0000
Glyma06G02990	AP3, ATAP3	1.1843	0.3381	0.1829	0.5857	0.0184	0.9298
Glyma06G10020	AGL22, SVP	-1.0975	0.0000	-0.9323	0.0000	-0.5349	0.0153
Glyma06G12380	PI	-Inf	0.3303	0.5329	0.1205	-0.2404	0.1033
Glyma06G22650	AGL8, FUL	1.0467	0.7784	0.2500	0.3758	-0.0356	0.8195
Glyma06G48270	AGL11, STK	NA	NA	1.0565	1.0000	-0.6807	0.0531
Glyma07G03401		NA	NA	NA	NA	-1.9364	0.6394
Glyma07G05001	AGL62	NA	NA	Inf	0.2309	2.4683	0.3962
Glyma07G05031	AGL62	NA	NA	NA	NA	Inf	0.9911
Glyma07G05061	AGL62	NA	NA	NA	NA	-Inf	0.7563
Glyma07G08831	AGL20, SOC1, ATSOC1	-0.4926	0.0413	0.0512	0.6775	-0.1097	0.5881
Glyma07G08890	AGL6	-0.3855	1.0000	0.3464	0.2411	0.1480	0.4155
Glyma07G14363	AGL44, ANR1	0.0727	1.0000	-0.2431	0.5124	-0.3081	0.4316
Glyma07G30040	AGL22, SVP	-0.4373	0.8807	-0.0365	0.7991	0.0765	0.7072
Glyma07G35610	AGL30	NA	NA	NA	NA	Inf	0.1763
Glyma08G03820	AGL91	-Inf	0.4849	-Inf	0.9983	0.0919	1.0000
Glyma08G03830	AGL29	-1.5473	0.5094	0.0536	1.0000	Inf	0.9911
Glyma08G06991	AGL11, STK	-Inf	0.1522	0.5424	0.2158	-0.3650	0.3530
Glyma08G07001	AGL21	0.8119	0.5170	-0.3461	0.4394	0.2421	0.5587
Glyma08G07260	AGL22, SVP	-0.0243	0.9620	-0.0124	0.8792	-0.0661	0.6113
Glyma08G10080		1.6216	0.7439	1.4480	0.3199	0.3908	0.9644
Glyma08G11110	AGL3, SEP4	-0.8101	0.0000	-0.1376	0.3348	0.1961	0.1913
Glyma08G11120	AGL9, SEP3	2.5015	0.1158	2.1449	0.0114	-0.2831	0.3798
Glyma08G12730	AG	-0.9778	1.0000	0.9970	0.0506	0.3442	0.1169
Glyma08G27670	AGL4, SEP2	NA	NA	0.4237	0.0461	-0.3249	0.0189
Glyma08G27680	AGL8, FUL	NA	NA	0.2771	0.3564	0.0676	0.7696
Glyma08G36380	AGL7, AP1	0.6216	0.9820	0.3635	0.0867	0.1812	0.2658
Glyma08G42300	AGL1, SHP1	-Inf	1.0000	0.5753	0.8484	0.2234	0.6020
Glyma09G11551	AGL38, PHE2	-Inf	1.0000	Inf	1.0000	-Inf	1.0000
Glyma09G27461	AGL6	NA	NA	-0.6478	0.1394	-0.2386	0.3928
Glyma09G33451	AGL21	-0.3891	0.4154	0.2776	0.5545	-0.3267	0.4666
Glyma09G36590	AGL12, XAL1	Inf	0.9928	Inf	0.7065	1.4138	0.6964
Glyma09G40230	AGL20, SOC1	-0.1103	0.5357	0.1058	0.2681	-0.0433	0.7802

Glyma09G40251	AGL6	NA	NA	NA	NA	1.2737	0.4800
Glyma09G42061	ABS, AGL32, TT16	-Inf	0.7046	NA	NA	-1.8982	0.4651
Glyma10G10295	AGL62	1.0558	0.6455	0.4249	0.5928	-0.8246	0.4429
Glyma10G11450	AGL62	NA	NA	Inf	1.0000	NA	NA
Glyma10G38541	AGL42	0.2470	0.9703	-0.2871	0.8060	-1.0187	0.5002
Glyma10G38580	AGL9, SEP3	NA	NA	0.5841	0.0394	-0.2656	0.1271
Glyma10G40060	AGL62	-Inf	0.7023	-Inf	0.9983	NA	NA
Glyma10G40070	AGL62	Inf	0.9928	NA	NA	Inf	0.9911
Glyma10G40080	AGL62	NA	NA	NA	NA	-Inf	1.0000
Glyma11G03261	AGL62	1.6420	0.7460	NA	NA	NA	NA
Glyma11G07820	AP3, ATAP3	-0.5814	0.4607	0.5595	0.0009	0.1914	0.5035
Glyma11G16105	AGL15	Inf	0.9928	0.2607	0.7146	-0.2725	0.6943
Glyma11G26260	AGL80, FEM111	NA	NA	-Inf	0.9983	NA	NA
Glyma11G30482	AGL80, FEM111	-Inf	1.0000	NA	NA	NA	NA
Glyma11G33460	AGL80, FEM111	0.1243	1.0000	Inf	0.7065	NA	NA
Glyma11G36890	AGL9, SEP3	4.1221	0.0067	0.5488	0.2695	-0.3005	0.2606
Glyma12G00770	AGL12, XAL1	-0.8757	1.0000	-1.8622	0.4511	0.2506	0.9501
Glyma12G13560	AP3, ATAP3	NA	NA	NA	NA	-Inf	1.0000
Glyma12G17721	AGL15	0.3479	0.5696	-0.0553	0.9880	0.0255	1.0000
Glyma13G02170	AGL104	NA	NA	NA	NA	2.8213	0.2615
Glyma13G06730	AGL4, SEP2	-0.7237	0.4782	0.7038	0.0002	0.2084	0.2272
Glyma13G06811	AGL8, FUL	2.0994	0.5546	0.5996	0.0229	-0.0776	0.7120
Glyma13G07720		-0.0932	0.7580	-0.0775	0.7312	-0.0209	0.9224
Glyma13G09660	PI	Inf	0.9928	0.3544	0.2691	0.0585	0.7852
Glyma13G29510	AG	1.0603	0.5484	0.8170	0.0089	0.2728	0.1464
Glyma13G32810	AGL21	-0.2157	0.4717	-0.2821	0.1694	-0.6063	0.0015
Glyma13G33031	AGL22, SVP	0.6453	0.8138	0.3936	0.1764	0.2296	0.4122
Glyma13G33051	AGL22, SVP	0.2478	0.5135	-0.3297	0.4691	0.1975	0.6425
Glyma14G03100	AGL5, SHP2	10.2290	0.0000	9.3020	0.0000	7.6001	0.0000
Glyma14G24590	PI	0.3271	0.5177	0.3580	0.0413	0.0749	0.6415
Glyma14G34160	AGL104	NA	NA	NA	NA	1.6326	0.2632

Glyma14G36221	AGL16	-0.2056	0.6147	-0.1984	0.6989	0.5145	0.2039
Glyma15G06302	AGL22, SVP	-0.3338	0.6678	-0.2445	0.4487	0.5634	0.0467
Glyma15G06314	AGL22, SVP	0.0328	0.9351	0.1483	0.4884	-0.0559	0.8159
Glyma15G06470	AGL44, ANR1	NA	NA	1.6357	0.7789	0.7583	0.6320
Glyma15G09500	AG	-0.8212	0.2399	0.1461	0.8522	-0.2370	0.2239
Glyma15G23335		0.6710	0.3237	-2.5343	0.5365	-0.6104	0.8753
Glyma15G23610	AP3, ATAP3	-1.5473	0.5094	-0.4578	0.9962	0.0823	1.0000
Glyma16G01541	AGL62	0.0222	1.0000	1.0507	1.0000	0.6641	0.9744
Glyma16G13070	AGL7, AP1	0.5214	0.7225	0.2736	0.0445	-0.0417	0.7314
Glyma16G17450	AP3, ATAP3	NA	NA	Inf	1.0000	Inf	0.7164
Glyma16G32540	AGL6	-Inf	1.0000	0.3458	0.4618	-0.3738	0.0903
Glyma17G08861	AGL3, SEP4	-Inf	0.7023	0.4407	0.1883	-0.1425	0.5368
Glyma17G08890	AGL8, FUL	-0.5383	0.8647	0.0820	0.4702	0.0202	0.8693
Glyma17G10941	AGL65	-1.3036	0.2895	1.5128	0.5062	0.5956	0.8272
Glyma17G14191	AGL19, GL19	-1.0822	0.0039	-0.8091	0.0367	-1.1492	0.2829
Glyma18G00801	AGL9, SEP3	NA	NA	0.6641	0.0664	-0.3180	0.0298
Glyma18G04760	AGL80, FEM111	-1.3010	0.3993	Inf	1.0000	0.3705	0.9602
Glyma18G05963	AGL80, FEM111	-Inf	0.7023	1.0565	1.0000	NA	NA
Glyma18G05976	AGL80, FEM111	NA	NA	NA	NA	Inf	0.9911
Glyma18G06040	AGL80, FEM111	-0.1700	1.0000	0.7472	0.5202	-0.2358	0.8506
Glyma18G12590	AGL1, SHP1	-Inf	0.7023	Inf	1.0000	0.2984	0.5325
Glyma18G33910	AP3, ATAP3	2.6924	0.1214	1.9278	0.0368	3.5377	0.0052
Glyma18G36263	AP3, ATAP3	Inf	0.9928	NA	NA	NA	NA
Glyma18G45761	AGL6	NA	NA	Inf	1.0000	-0.7264	0.5776
Glyma18G45780	AGL20, SOC1 ATSOC1	-0.1081	0.3017	0.1282	0.1430	-0.1346	0.2294
Glyma18G50900	AGL4, SEP2	2.4264	0.4082	0.7034	0.0070	0.1493	0.4361
Glyma18G50910	AGL8, FUL	-0.5734	0.2259	-0.0824	0.7467	0.3363	0.2750
Glyma19G04320	AGL4, SEP2	Inf	0.1370	0.4935	0.0188	0.0559	0.8244
Glyma19G04326	AGL10, CAL, CAL1	Inf	0.9928	0.1751	0.7200	0.4812	0.1763
Glyma19G06145		-0.9778	1.0000	0.0211	1.0000	-0.2776	1.0000

Glyma19G06498		NA	NA	Inf	1.0000	-0.0027	1.0000
Glyma19G07162		Inf	0.9928	NA	NA	0.0836	1.0000
Glyma20G00401	ABS, AGL32, TT16	NA	NA	-Inf	0.9983	-2.7236	0.3021
Glyma20G04500	AGL30	NA	NA	NA	NA	2.4126	0.0903
Glyma20G27320	AGL62	Inf	0.9928	NA	NA	NA	NA
Glyma20G27341	AGL62	-0.8757	1.0000	-Inf	0.9983	NA	NA
Glyma20G27361	AGL61, DIA	NA	NA	NA	NA	1.0358	0.9812
Glyma20G29250	AGL9, SEP3	NA	NA	0.9264	0.0029	0.1626	0.3639
Glyma20G29300	AGL42	Inf	0.4607	0.7421	0.1631	-0.2370	0.7084

Data listed the ID of genes which significant regulated in *GmAGL1*-OX transgenic plants, their corresponding homologous genes of Arabidopsis, the$\log_2$ Fold Change ($\log_2$FC) and P value in VC, V4 and R1 period. NA indicate the gene is no expression. “Inf” indicate the gene express in transgenic plants but not in WT. “– Inf” indicate the gene express in WT but not in transgenic plants.

Supporting Table 4. Transcripts associated with vernalization pathway.

Gene ID	At Ortholog	VC		V4		R1	
		log2FC	P value	log2FC	P value	log_2FC	P value
Glyma01G11755	VRN1	NA	NA	NA	NA	NA	NA
Glyma04G43620	VRN1	-0.9459	0.2994	-0.0548	0.8783	-0.2036	0.5895
Glyma07G19395	VRN1	NA	NA	NA	NA	-Inf	0.2865
Glyma07G21160	VRN1	0.1430	0.2492	-0.1998	0.0974	-0.0516	0.7163
Glyma08G44640	VRN1	-0.6487	0.7482	0.3242	0.8800	0.3269	0.7126
Glyma08G44655	VRN1	NA	NA	NA	NA	NA	NA
Glyma09G18790	VRN1	0.7825	0.7996	0.1536	0.6819	-0.5633	0.1652
Glyma09G20150	VRN1	NA	NA	- Inf	0.7018	NA	NA
Glyma09G20281	VRN1	0.1834	0.9622	1.6386	0.5092	0.6899	0.5476
Glyma10G42762	VRN1	NA	NA	NA	NA	NA	NA
Glyma10G42774	VRN1	0.0467	1.0000	-0.0693	1.0000	1.0823	0.7924
Glyma10G42787	VRN1	-0.3650	1.0000	0.0507	1.0000	2.0167	0.5926
Glyma11G13210	VRN1	0.0928	0.5273	0.0480	0.6932	0.0100	0.9224
Glyma11G13220	VRN1	-0.2407	0.4845	0.9905	0.2928	0.3736	0.3895
Glyma11G13231	VRN1	0.9947	0.7980	- Inf	0.9983	0.3872	0.5349
Glyma12G05250	VRN1	0.2090	0.2392	0.0133	0.9532	-0.0375	0.7917
Glyma14G08641	VRN1	-0.0504	0.7690	0.0371	0.8197	-0.0712	0.6620
Glyma16G05110	VRN1	0.2499	0.8561	0.2182	0.6264	-0.4615	0.2948
Glyma18G30790	VRN1	-0.1303	0.9082	0.2447	0.4181	-0.0215	0.9508
Glyma19G27955	VRN1	NA	NA	NA	NA	NA	NA
Glyma20G01130	VRN1	-0.0632	0.5851	0.0156	0.9021	0.0287	0.8314
Glyma20G24211	VRN1	NA	NA	NA	NA	Inf	0.9911
Glyma20G24222	VRN1	Inf	0.9928	- Inf	0.9983	-1.9081	0.6416
Glyma20G24244	VRN1	NA	NA	NA	NA	NA	NA
Glyma20G24268	VRN1	NA	NA	Inf	1.0000	NA	NA
Glyma19G41287	VRN2	-0.5288	0.5719	0.0229	1.0000	-0.0672	0.9713
Glyma05G35280	VRN5	0.0372	0.7162	-0.1470	0.2268	-0.0558	0.6581
Glyma07G09800	VRN5	-0.2154	0.0824	-0.2517	0.0131	0.1534	0.2249
Glyma08G04440	VRN5	-0.0416	0.7989	-0.0561	0.7573	0.0337	0.8074

Glyma09G32010	VRN5	-0.0431	0.6496	-0.0191	0.8566	0.0958	0.4370
Glyma10G40631	RTV1	NA	NA	- Inf	0.9983	1.4645	0.6397
Glyma02G37880	VEL1	0.0625	0.6004	0.1434	0.2632	0.0874	0.5631
Glyma13G00920	VEL1	-0.0899	0.2567	-0.2379	0.0226	0.0520	0.6837
Glyma14G36100	VEL1	0.0859	0.5300	0.0249	0.8900	0.1221	0.4715
Glyma17G07000	VEL1	-0.0221	0.7704	-0.2133	0.0374	0.1521	0.2299
Glyma08G21490	ELF7	0.1212	0.1827	-0.0189	0.8632	-0.0866	0.4823
Glyma07G01830	ELF7	0.0174	0.8689	-0.0003	1.0000	0.0525	0.6905
Glyma09G07980	ELF8	-0.0057	0.9431	-0.1478	0.1221	-0.2829	0.0130
Glyma15G19450	ELF8	-0.0196	0.8607	-0.1003	0.3266	-0.1732	0.1542
Glyma13G31033	FES1	0.0686	0.5813	-0.1367	0.2324	0.0010	0.9802
Glyma15G08331	FES1	0.1624	0.1983	-0.0171	0.9064	-0.1925	0.1834

Data listed the ID of genes which significant regulated ($P < 0.05$) in *GmAGL1*-OX transgenic plants, with their corresponding homologous genes of Arabidopsis, the $\log_2$ Fold Change ($\log_2$FC) and P value in VC,V4 and R1 period. NA indicate the gene is no expression. "Inf" indicate the gene express in transgenic plants but not in WT. "– Inf" indicate the gene express in WT but not in transgenic plants.

Supporting Table 5. Transcripts associated with the autonomous pathway

Gene ID	At Ortholog	VC		V4		R1	
		log2FC	P value	log2FC	P value	log2FC	P value
Glyma02G29380	PIE1	-0.1383	0.1786	0.0716	0.4848	-0.1538	0.2157
Glyma05G24002	PIE1	1.3584	0.6102	1.5879	0.1900	0.3180	0.9604
Glyma09G17220	PIE1	-0.0113	0.9165	-0.0527	0.5771	-0.1622	0.1659
Glyma04G38071	FRI	-0.1181	0.3081	-0.0814	0.5326	0.0960	0.5066
Glyma06G17005	FRI	0.1806	0.1124	-0.1190	0.3431	-0.0285	0.8569
Glyma01G07320	FIP1	NA	NA	NA	NA	NA	NA
Glyma09G08090	FIP1	0.0122	0.9690	0.1399	0.3983	0.0641	0.7220
Glyma15G19570	FIP1	-0.0621	0.6363	-0.0316	0.7982	0.0166	0.9116
Glyma16G28370	VIP1	0.0023	1.0000	0.1421	0.6526	0.2552	0.2426
Glyma16G28385	VIP1	0.0699	1.0000	NA	NA	1.1311	0.9810
Glyma02G09140	VIP1	-0.1191	0.4206	0.1050	0.5664	-0.0013	0.9970
Glyma02G09147	VIP1	NA	NA	NA	NA	Inf	0.9911
Glyma02G09162	VIP1	Inf	0.9928	NA	NA	NA	NA
Glyma13G16700	VIP3	-0.0103	0.9529	-0.1893	0.1749	0.0186	0.9126
Glyma17G05990	VIP3	0.0416	0.6114	-0.0977	0.3337	0.0857	0.5027
Glyma04G32540	VIP4	-0.0392	0.6264	-0.0830	0.3657	-0.0046	0.9937
Glyma07G31365	VIP5	0.0707	0.5067	-0.0429	0.7109	0.0904	0.4760
Glyma13G05760	VIP5	-0.2021	0.3518	-0.1112	0.5077	-0.1909	0.2853
Glyma13G25093	VIP5	0.1061	0.3004	-0.0728	0.4962	0.0665	0.5724
Glyma19G35350	VIP5	0.6090	0.4348	-0.1741	0.7426	-0.0448	0.9750
Glyma03G32600	VIP5	0.0263	1.0000	-0.1748	0.6489	0.0045	1.0000
Glyma03G38980	VIP5	-0.9301	1.0000	NA	NA	-0.8064	0.4411
Glyma05G25030	FCA	1.1408	0.2990	NA	NA	Inf	0.9911
Glyma07G36630	FCA	0.0503	0.5363	-0.2567	0.0086	0.0723	0.5230
Glyma03G37950	FCA	0.0984	0.5225	0.1299	0.3296	-0.0690	0.6653
Glyma17G03960	FCA	0.0457	0.6521	-0.0256	0.8091	-0.1296	0.2807
Glyma19G40570	FCA	-0.0397	0.7856	0.0501	0.6967	-0.2017	0.2102
Glyma13G26820	FY	0.1168	0.2167	-0.1146	0.3080	-0.1146	0.3080
Glyma15G37830	FY	0.1251	0.3003	-0.1555	0.2200	-0.1555	0.2200

Glyma02G18610	FLD	0.2008	0.0701	0.0533	0.6613	0.0150	0.8936
Glyma09G07120	FVE	0.0701	0.3032	-0.0406	0.6262	-0.0808	0.4319
Glyma13G42660	FVE	0.1515	0.2636	-0.1980	0.0840	0.0324	0.8217
Glyma15G02770	FVE	-0.4874	0.0100	-0.1592	0.4072	0.3160	0.0788
Glyma15G18450	FVE	-0.0372	0.6094	0.0049	0.9496	-0.0574	0.5893
Glyma03G36970	LD	-0.0633	0.6401	-0.1700	0.1494	-0.1204	0.4037
Glyma19G39620	LD	-0.0453	0.7177	-0.0804	0.4977	-0.0379	0.8190
Glyma13G42061	FPA	-0.0880	0.4763	0.2375	0.0405	-0.1973	0.2367
Glyma15G03335	FPA	-0.0487	0.7361	0.1762	0.1026	-0.0187	0.9142
Glyma11G13490	FPA	0.0398	0.6786	0.1535	0.1384	-0.2889	0.0156
Glyma12G05490	FPA	0.1759	0.3326	0.0331	0.9255	0.1286	0.5595

Data listed the ID of genes which significant regulated ($P < 0.05$) in *GmAGL1*-OX transgenic plants, with their corresponding homologous genes of Arabidopsis, the $\log_2$ Fold Change ($\log_2$FC) and P value in VC,V4 and R1 period. NA indicate the gene is no expression. "Inf" indicate the gene express in transgenic plants but not in WT. "– Inf" indicate the gene express in WT but not in transgenic plants.

Supporting Table 6. Transcripts associated with the GA pathway.

Gene ID	At Ortholog	VC		V4		R1	
		log2FC	P value	log2FC	P value	log2FC	P value
Glyma20G34260	GAI	0.3220	0.2788	0.4578	0.0109	0.2174	0.2580
Glyma04G21340	GAI	0.2739	0.0311	0.2371	0.1689	-0.0725	0.7428
Glyma10G02790	GID1B	-0.0432	0.8442	0.7690	0.0000	0.0788	0.5445
Glyma19G33381	GID1B	-0.0010	1.0000	0.1388	0.3733	0.1136	0.5475
Glyma02G17010	GID1B	0.1418	0.5515	0.7078	0.0000	0.0242	0.8888
Glyma03G30460	GID1B	0.1922	0.4790	0.7690	0.0000	-0.3216	0.1668
Glyma10G29910	GID1C	-0.0888	0.3798	0.1326	0.1773	0.1384	0.2380
Glyma13G25900	GID1C	-0.2016	0.2554	-0.2217	0.3891	0.0188	0.9890
Glyma20G37430	GID1C	-0.2243	0.0660	0.3362	0.0006	-0.1233	0.4315
Glyma06G47000	MYB65	0.0138	1.0000	-0.1940	0.6230	0.0928	0.8370
Glyma02G01530	RGA1	0.0254	0.7653	-0.2316	0.0155	Inf	0.9911
Glyma05G27190	RGA1	0.1503	0.2604	0.2274	0.1219	0.2599	0.0690
Glyma06G23940	RGA1	-0.1008	0.1954	-0.2952	0.0027	-0.2817	0.0825
Glyma08G10140	RGA1	0.1218	0.5841	0.0800	0.6504	-0.1287	0.2631
Glyma10G33380	RGA1	0.0254	0.7653	-0.2316	0.0155	-0.1595	0.3225
Glyma05G03020	RGL1	-0.0538	0.8779	2.3421	0.0033	0.1072	0.8558
Glyma08G25800	RGL1	Inf	0.9928	NA	NA	- Inf	1.0000
Glyma17G13680	RGL1	Inf	0.4571	1.5743	0.5077	-0.2458	0.8170
Glyma18G43580	RGL1	NA	NA	NA	NA	-2.7274	0.0000
Glyma19G40440	RGL1	-1.9032	0.5875	0.9278	1.0000	0.0358	1.0000
Glyma09G04110	RGL2	1.0222	0.9863	-1.3524	0.2517	0.8573	0.4814
Glyma10G01571	RGL2	Inf	0.6853	-0.6164	0.9967	-0.4930	1.0000
Glyma11G33720	RGL2	0.0763	0.2966	0.0976	0.2588	0.0574	0.5757
Glyma15G15110	RGL2	-0.9397	0.8179	2.1602	0.2370	0.1898	0.9321
Glyma18G04500	RGL2	-0.0192	0.7865	0.0485	0.5880	0.0383	0.6958
Glyma03G37851	RGL3	-1.4893	0.0443	0.0565	1.0000	- Inf	1.0000

Data listed the ID of genes which significant regulated ($P < 0.05$) in *GmAGL1*-OX transgenic plants, with their corresponding homologous genes of Arabidopsis, the $\log_2$ Fold Change ($\log_2$FC) and P value in VC,V4 and R1 period. NA indicate the gene is no expression. “Inf” indicate the gene express in transgenic plants but not in WT. “– Inf” indicate the gene express in WT but not in transgenic plants.
