# Supplementary figures and images for: Soybean MADS-box gene GmAGL1 promotes flowering via the photoperiod pathway
Source: BMC Genomics. 2018 Jan 16;19:51. doi: 10.1186/s12864-017-4402-2 (PMC5769455; doi:10.1186/s12864-017-4402-2)

WT

Transgenic

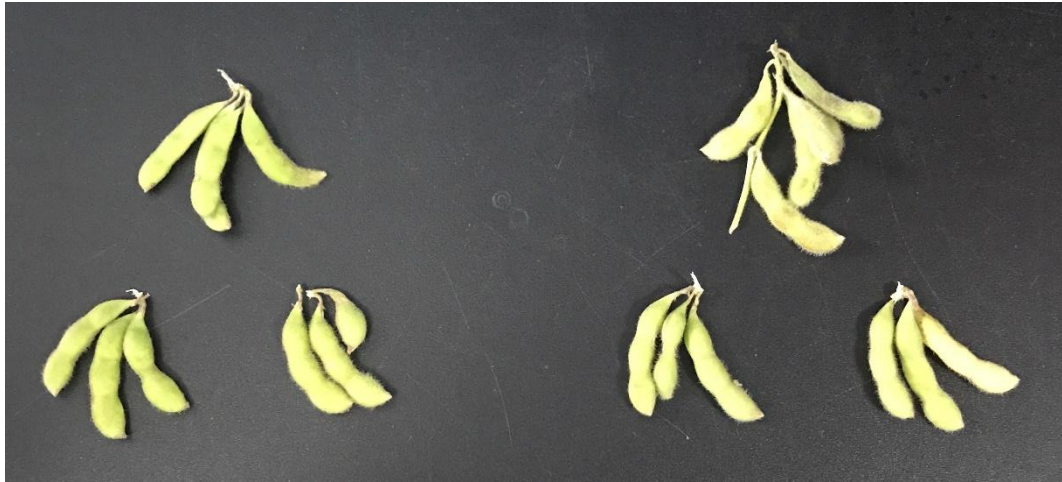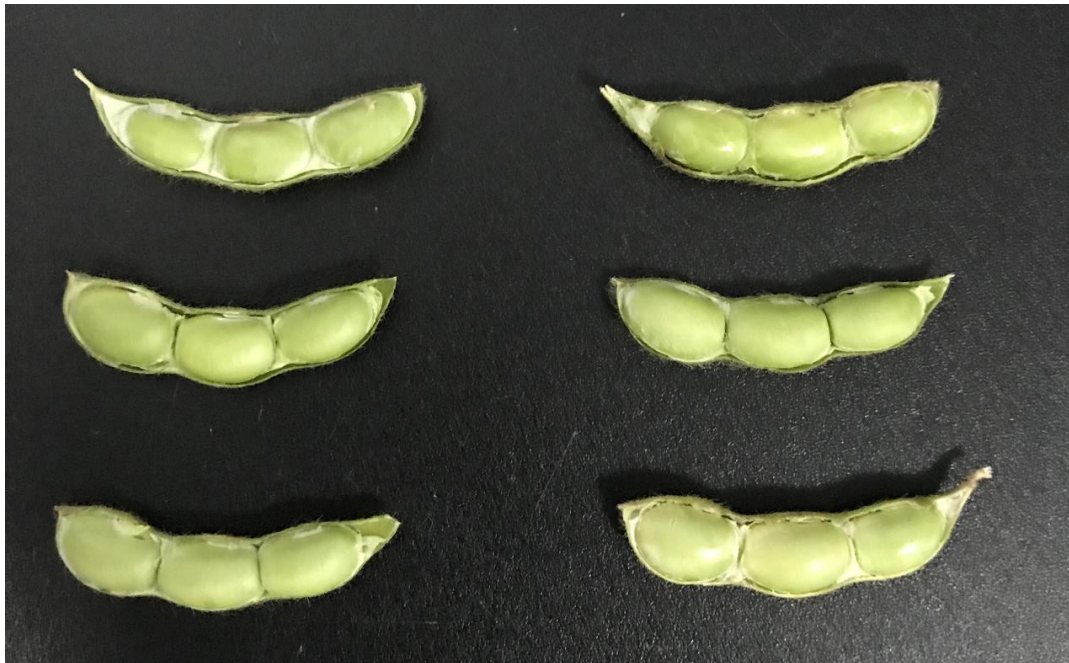

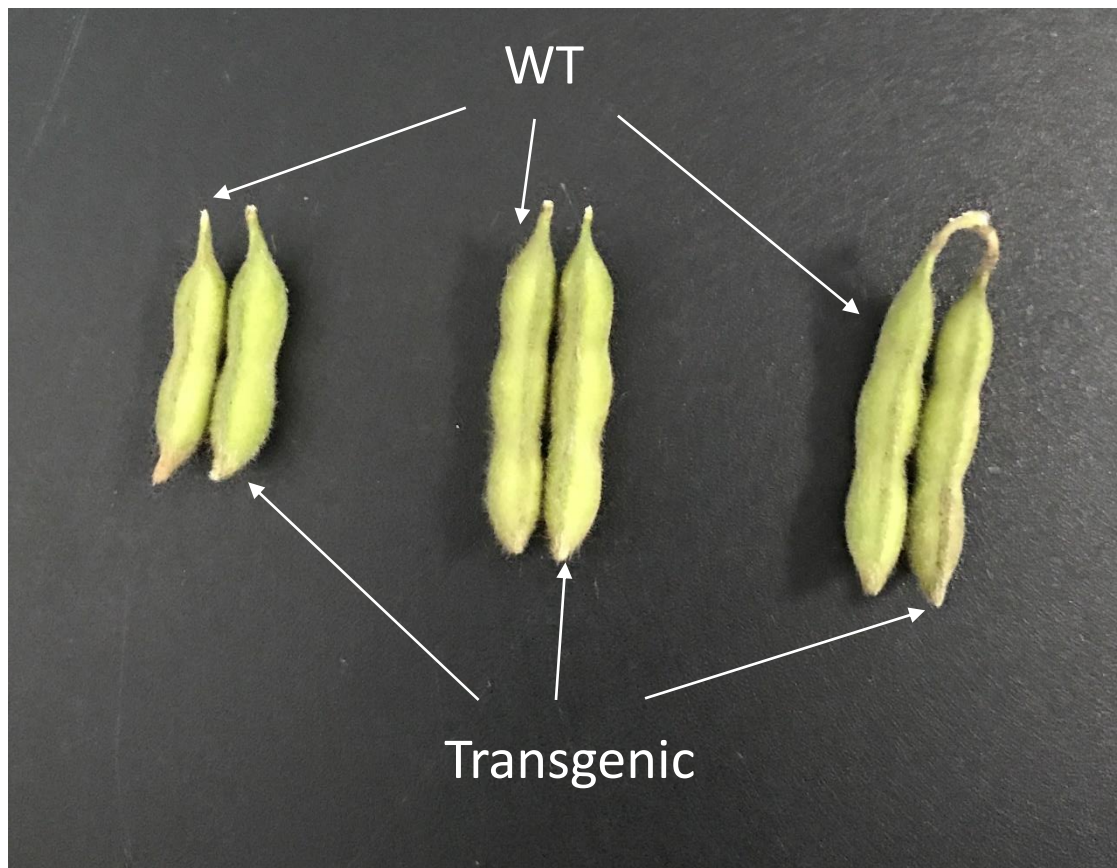

Supplement: Supplementary file 3 — Phenotype of immature pods. (PDF 427 kb) [file 12864_2017_4402_MOESM3_ESM.pdf]
