# Supplementary material for: Soybean MADS-box gene GmAGL1 promotes flowering via the photoperiod pathway
Source: BMC Genomics. 2018 Jan 16;19:51. doi: 10.1186/s12864-017-4402-2 (PMC5769455; doi:10.1186/s12864-017-4402-2)

Experimental process of GmAGL1 transformation

- Method: Agrobacterium - mediated cotyledon method
- Marker gene：cp4-EPSPS
- Screening agent: glyphosate 5-10mg / L
- Plant Material：Jack

Experimental steps

1、 Selected soybean seeds

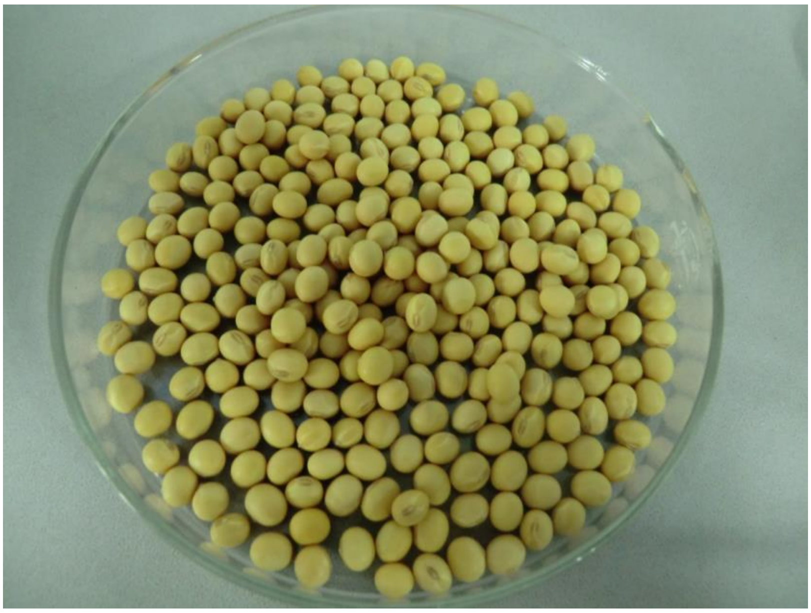

2、 Seeds disinfection

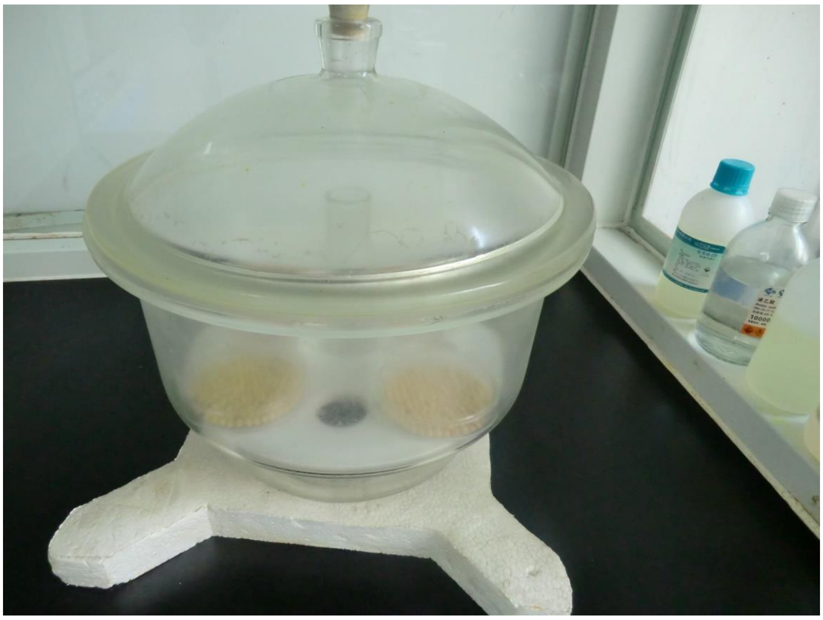

3、 Seeds germination

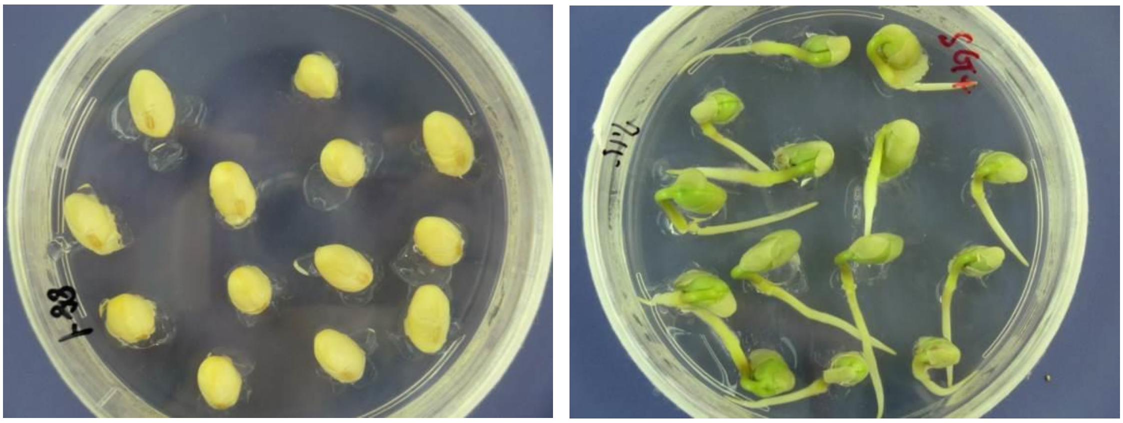

4、 Agrobacterium infection of soybean cotyledon

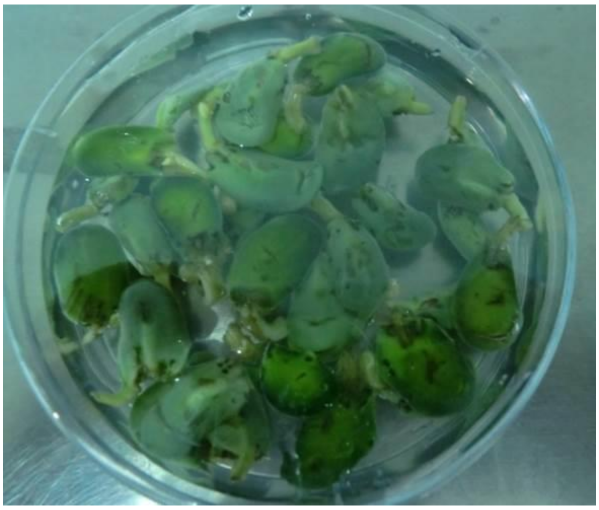

5、 Agrobacterium and soybean explants co-culture

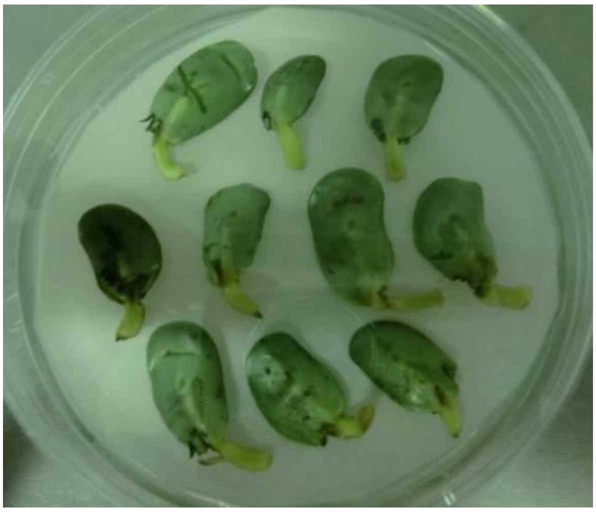

6、 Shoots induction

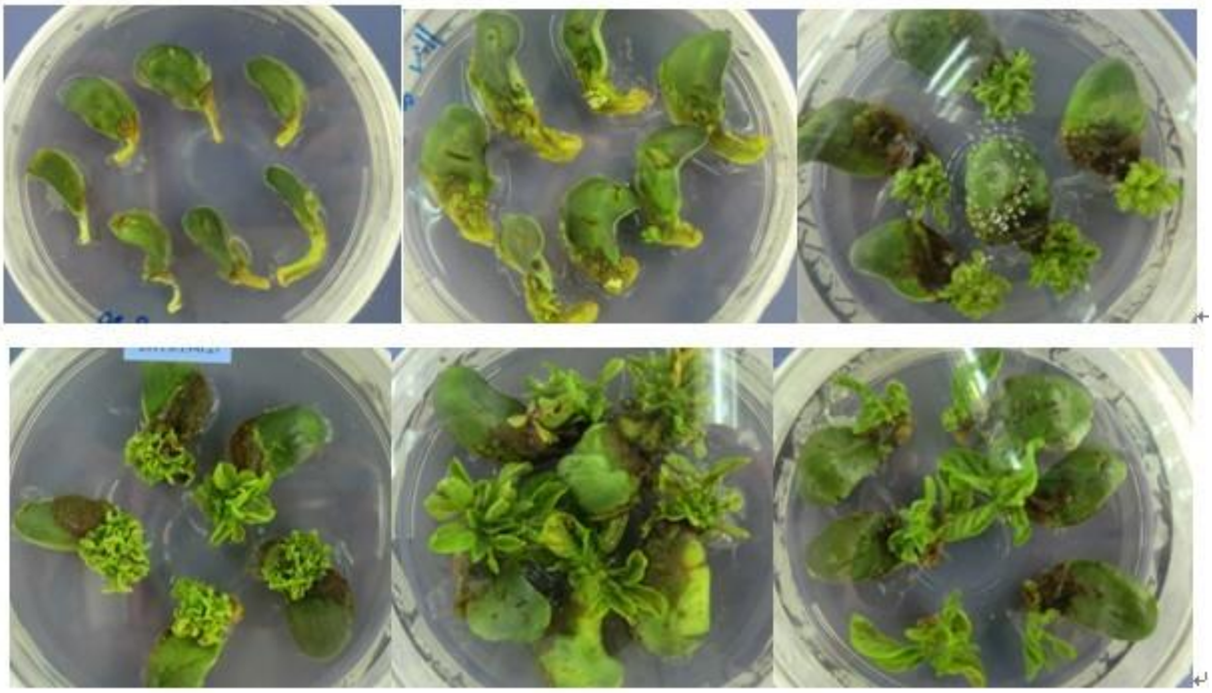

7、 Shoots elongation

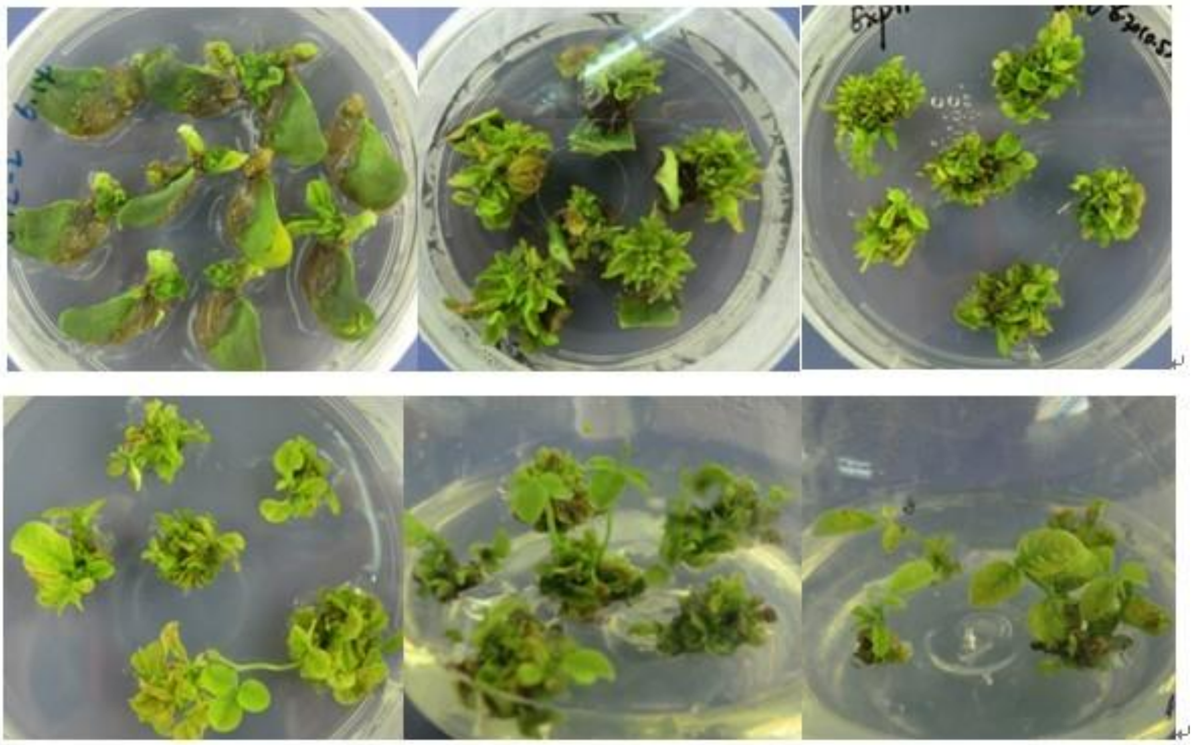

8、 Tissue culture seedlings rooting

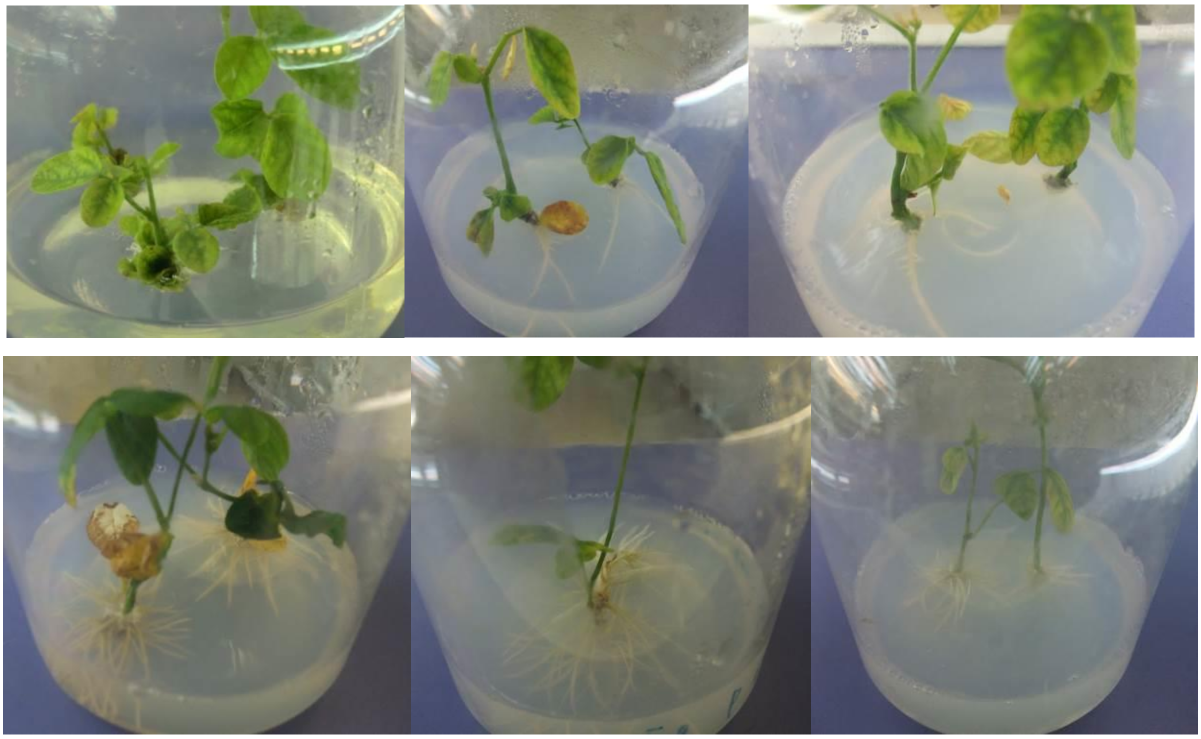

9、 Tissue culture plants indoor transplantion

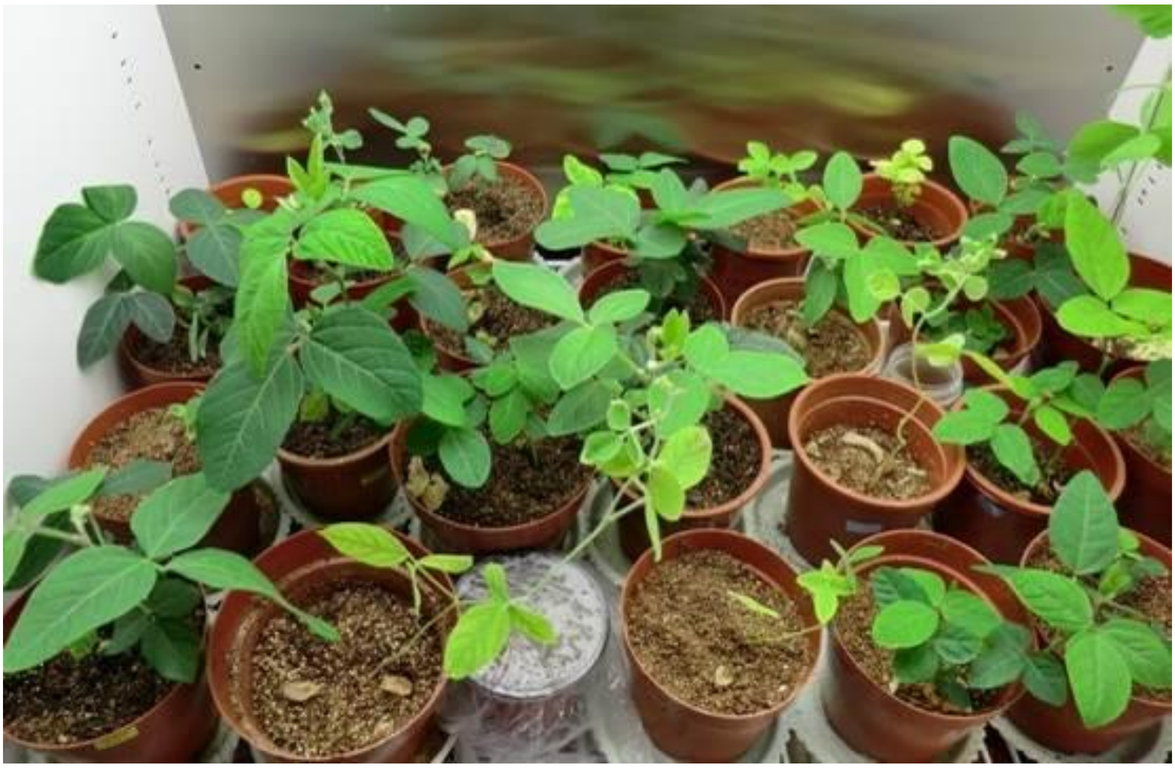

10、 Pods of transgenic plants

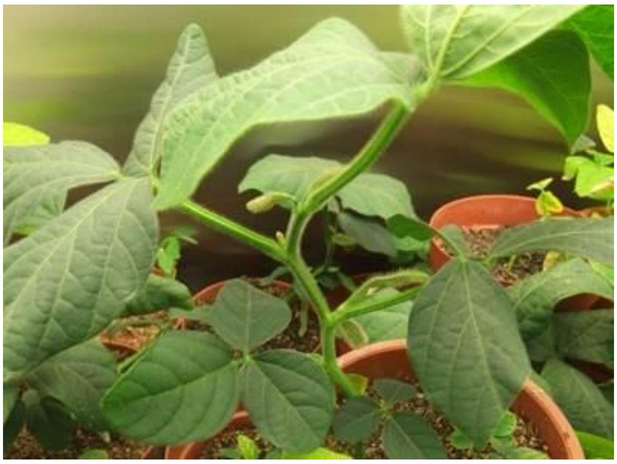

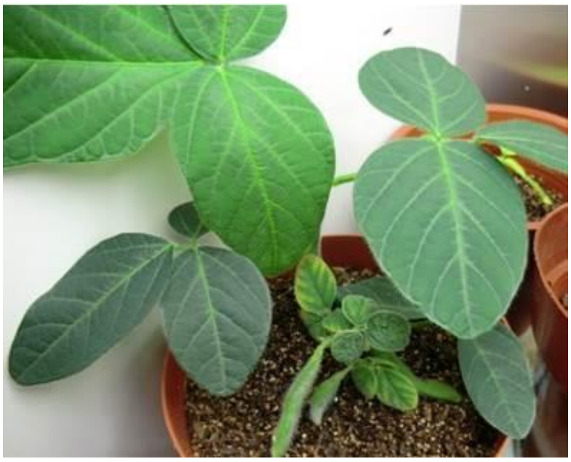

11、 Detection of T0 generation transgenic plants

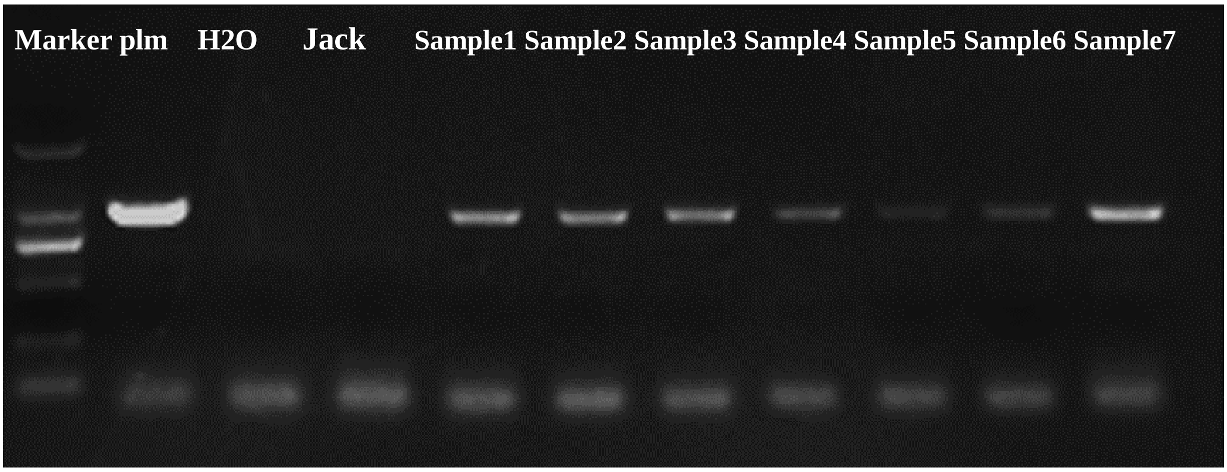

Supplement: Supplementary file 4 — The growth of transgenic plants in different periods. (PDF 560 kb) [file 12864_2017_4402_MOESM4_ESM.pdf]
